# Ligand-Directed Self-Assembling Chimeras for Targeted Protein O‑GlcNAcylation

**DOI:** 10.1021/acschembio.5c00684

**Published:** 2025-12-05

**Authors:** Zhihao Guo, Tongyang Xu, Khadija Shahed Khan, Stephan Scheeff, Yao Qin, Sin-Yi Yu, Richard Lo, Yuanpei Li, Yalun Xie, Bowen Ma, Yunpeng Huang, Hillary Yui-Yan Yip, Clive Yik-Sham Chung, Tomonori Tamura, Itaru Hamachi, Billy Wai-Lung Ng

**Affiliations:** 1 Guangdong-Hong Kong-Macao Joint Laboratory for New Drug Screening, School of Pharmacy, 26451 The Chinese University of Hong Kong, Sha Tin, Hong Kong; 2 School of Biomedical Sciences, Faculty of Medicine, 26451 The Chinese University of Hong Kong, Sha Tin, Hong Kong; 3 School of Biomedical Sciences, Li Ka Shing Faculty of Medicine, 25809 The University of Hong Kong, Pok Fu Lam, Hong Kong; 4 Department of Synthetic Chemistry and Biological Chemistry, Graduate School of Engineering, 12918 Kyoto University, Katsura, Kyoto 615-8510, Japan; 5 Li Ka Shing Institute of Health Sciences, Faculty of Medicine, 26451 The Chinese University of Hong Kong, Sha Tin, Hong Kong; 6 Gerald Choa Neuroscience Institute, 26451 The Chinese University of Hong Kong, Sha Tin, Hong Kong; 7 Peter Hung Pain Research Institute, Faculty of Medicine, 26451 The Chinese University of Hong Kong, Sha Tin, Hong Kong

## Abstract

Precise control of protein-specific O-GlcNAcylation in cells remains a major challenge. Chemically induced proximity (CIP) offers a promising path forward, but its application to targeted protein O-GlcNAcylation has been limited by the lack of ligands that can bind the O-GlcNAc transferase (OGT) without inhibiting its catalytic function. Here, we repurpose a potent OGT inhibitor into a noninhibitory covalent probe using ligand-directed release chemistry (LDR). The resulting ligands covalently label OGT while preserving its enzymatic activity. Building on this scaffold, we developed a self-assembling O-GlcNAcylation Targeting Chimera (OGTAC) that recruits OGT to its native substrate casein kinase IIα (CK2α) in living cells, selectively elevating CK2α O-GlcNAcylation without affecting global modification levels. This new class of self-assembling chimeras covalently engages OGT to induce protein-specific O-GlcNAcylation, offering a versatile platform for dissecting and controlling this essential modification in living cells. Our findings open the door to next-generation OGTACs and related therapeutic strategies for the targeted modulation of the O-GlcNAc signaling.

## Graphical abstract

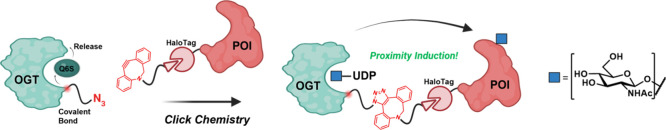

## Introduction

O-Linked *N*-acetylglucosamine protein modification (O-GlcNAcylation) is a dynamic post-translational modification (PTM) in which a single *N*-acetylglucosamine moiety is attached to serine or threonine residues of nuclear, cytoplasmic, and mitochondrial proteins.^1–3^ This modification regulates diverse processes, including transcription,^3^ signaling,^4^ and protein localization,^5,6^ and its dysregulation is associated with cancer, diabetes, cardiovascular disease, and neurodegenerative disorders.^4,7–10^ Of note, O-GlcNAcylation is uniquely controlled by two enzymes—O-GlcNAc transferase (OGT)^11^ and O-GlcNAcase (OGA).^12^

To dissect the site-specific functional consequences of O-GlcNAcylation, several pioneering strategies have been developed. Early work by the Pratt group utilized synthetic O-GlcNAcylated peptides to demonstrate that this modification could inhibit the peptide-dependent acceleration of α-synuclein aggregation.^13^ To achieve site-specific modification of full-length proteins, the same group later employed sophisticated expressed protein ligation (EPL) to incorporate O-GlcNAc or its enzymatically stable mimic S-GlcNAc, enabling precise biochemical studies *in vitro*.^14^ In parallel, a genetic recoding strategy was established by the Daan van Aalten group, which exploits the promiscuity of the OGT to install the hydrolysis-resistant S-GlcNAc mimic at specific sites by mutating serine or threonine to cysteine, enabling stable, site-specific mimicry of the O-GlcNAcylation in mammalian cells using CRISPR-Cas9 genome editing.^15,16^ Additionally, the Woo group established a genetic strategy using nanobody-mediated recruitment of OGT to achieve protein-specific O-GlcNAcylation *in cellulo* ^17^ and the Hart group developed a dual RNA-aptamer system that recruited OGT to β-catenin, demonstrating the utility of induced proximity for targeted O-GlcNAcylation in cells.^18^ While powerful, these strategies have limitations—most notably their reliance on genetic manipulation—which poses challenges for cell permeability and *in vivo* delivery. These constraints underscore the need for alternative approaches based on cell-permeable small molecules that can ideally target endogenous proteins directly.

Chemical inducers of proximity (also known as bifunctional molecules) have emerged as powerful tools to manipulate PTMs with high selectivity by bringing a modifying enzyme into proximity with a chosen protein substrate.^19,20^ Such strategies can up- or down-regulate specific PTMs while minimizing effects on the global proteome. Previous examples include PROTACs^21^ for targeted protein degradation, AceTAGs^22^ for acetylation, PHICs^23^ for phosphorylation, and MrTACs^24^ for methylation. More recently, our laboratory pioneered the development of the first-generation O-GlcNAcylation-targeting chimeras (OGTACs).^25^ OGTACs can recruit engineered FKBP12^F36V^-tagged OGT to specific substrates, thereby selectively increasing their O-GlcNAcylation in cells.^25,26^ While this proof-of-concept validated the OGTAC strategy, reliance on engineered OGT limits physiological relevance and potential translational applications. Therefore, developing OGTACs that can recruit an endogenous OGT is crucial; however, progress has been limited by the lack of noninhibitory OGT ligands. Most reported small-molecule OGT ligands are potent inhibitors,^27–33^ which, if used as recruitment handles, would compromise the catalytic activity of OGT and impair O-GlcNAcylation induction. This bottleneck is reminiscent of the early PROTAC field, where the absence of suitable E3 ligase recruiters stalled development for nearly a decade.^34^

Here, we address this challenge by re-engineering the potent OGT inhibitor^29^ OSMI-4 into a noninhibitory covalent probe using ligand-directed release chemistry (LDR).^35–40^ Incorporating an *N*-acyl-*N*-alkyl sulfonamide (NASA)^37^ warhead enabled covalent labeling of OGT while preserving its catalytic function. We then leveraged this ligand to construct an in-cell self-assembling OGTAC system, which induces the proximity between OGT and a protein of interest (POI)—depicted here with CK2α—to achieve selective O-GlcNAcylation without altering global modification levels. This strategy allows for the direct interrogation of how POI-specific alterations in O-GlcNAcylation affect protein function, circumventing the confounding influence of global O-GlcNAc perturbation. Our study demonstrates the feasibility of chemically engaging OGT for programmable, protein-specific O-GlcNAcylation, laying a foundation for next-generation OGTACs and therapeutic strategies targeting O-GlcNAc signaling.

## Result

### LDR Chemistry Converts an OGT Inhibitor into a Noninhibitory Covalent Ligand

Our group pioneered first-generation OGTACs,^25^ establishing proof-of-concept for targeted O-GlcNAcylation using a chemogenetic approach (Figure 1a). To advance this platform, we ultimately aim to develop OGTACs that can engage endogenous OGT, thereby enhancing druglikeness and broadening applicability across biological contexts. In this study, we report the first step toward this goal (Figure 1a).

**Figure 1:**
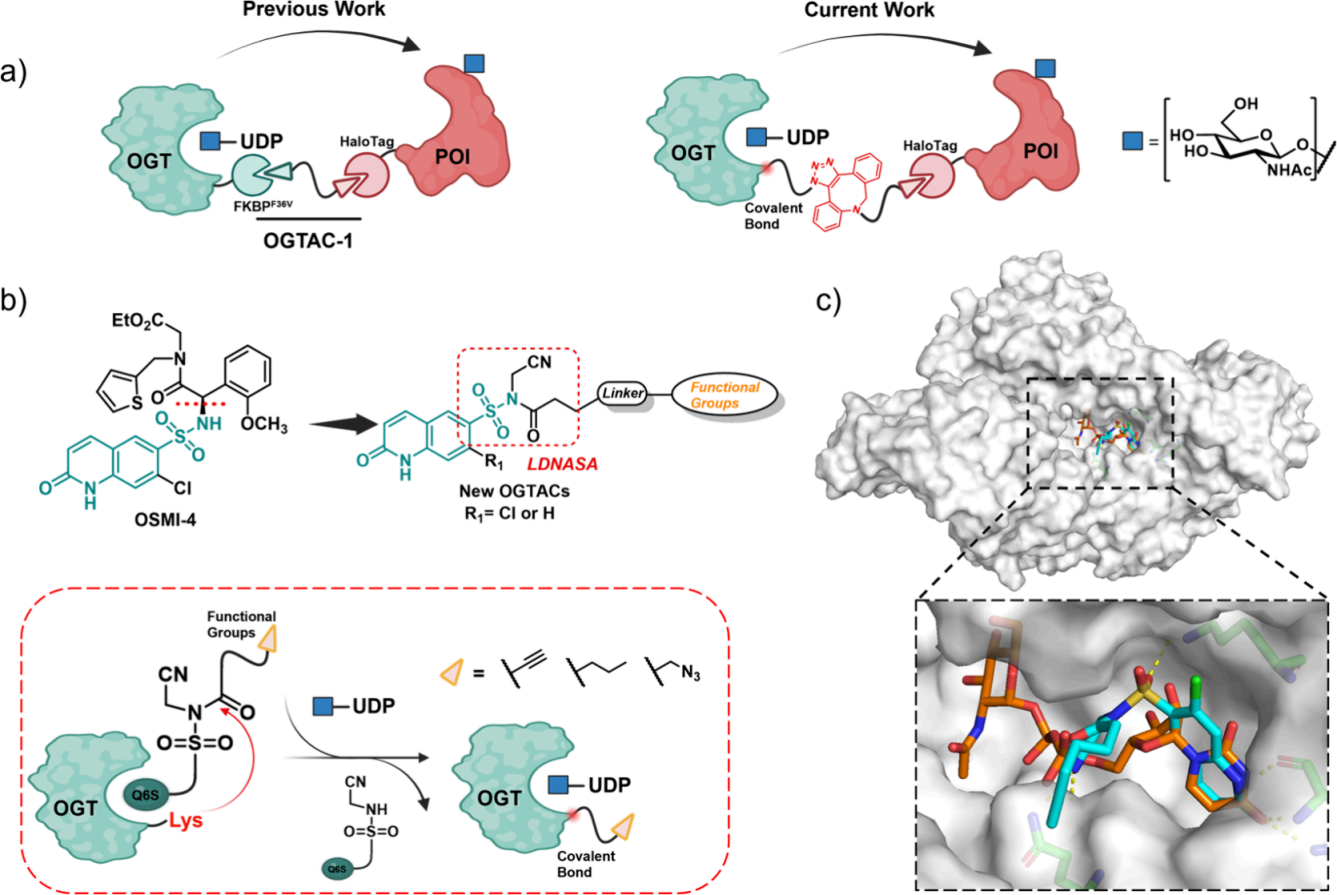
OSMI-4 scaffold re-engineered with a NASA warhead to label OGT while preserving its catalytic activity. (a) Schematic illustration of OGTACs. (b) Design of new OGTACs based on truncated OSMI-4 through LDR. Green color denotes the Q6S core. (c) An overlay of truncated OSMI-4 (cyan) with UDP-GlcNAc (orange) confirms that the Q6S group binds to OGT (PDB: 4N3C).

We focus on OSMI-4, the most potent OGT inhibitor reported to date,^41^ whose quinolinone-6-sulfonamide (Q6S) moiety mimics uracil that occupies the OGT catalytic pocket.^29^ The U-shaped conformation formed by the S–N bond optimally occupies the pocket, representing a key prerequisite for the inhibitory activity of this compound class.^27,29^ Further potency and selectivity enhancements were achieved through the phenylalanine group and the ortho-chloro substituent on the sulfonamide ring, which strengthen interactions with the pocket.^29^ For the design of a bifunctional molecule, we simplify the OSMI-4 scaffold to minimize inhibitory interactions, retaining only the essential Q6S core (Figure 1b). A linker and functional group are introduced at the sulfonamide nitrogen, concurrently incorporating a cyano group to create a ligand-directed NASA warhead. This warhead reacts rapidly with the amino group of lysine residues, forming a covalent bond.^37,40^ Subsequent dissociation of the Q6S moiety leaves the functional group or protein ligand tethered. This design allows the recruitment of a second protein to the OGT, bringing it into proximity and thereby selectively enhancing the O-GlcNAcylation of that target POI (Figure 1a). Furthermore, docking simulations confirm that the simplified molecules retain the ability to mimic uridine and engage in the OGT binding pocket (Figure 1c).

### Modular Synthetic Route Delivers Tunable OGT Probes

To obtain a panel of covalent OGT probes for evaluation, we established a concise modular synthetic route (Scheme S1). Starting from commercially available 2-quinolinol or 7-chloro-2-hydroxyquinoline, chlorosulfonation under reflux yielded the key 6-sulfonyl chloride intermediates **2a**,**b**.^29^ Subsequent reaction of the crude product with aqueous ammonia, also under reflux, provided the 6-sulfonamide intermediates **3a**,**b**,^42^ which precipitated from the reaction mixture and were isolated directly by filtration. Coupling of these sulfonamides with carboxylic-acid-containing side chains, mediated by EDCI in the presence of DMAP and DIPEA, furnished key intermediates **5a**–**c** and **6a**,**b**. Finally, substitution of the sulfonamide N–H proton with a cyanomethyl group under basic conditions (DIPEA) yielded the target compounds with the Q6S moiety^37^ (Table 1). This flexible route provided multiple probe analogues for the subsequent testing of the OGT labeling and the OGTAC assembly.

**Table 1:**
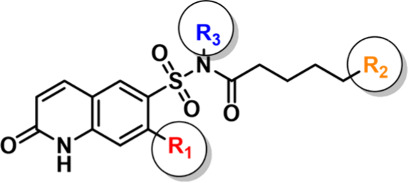

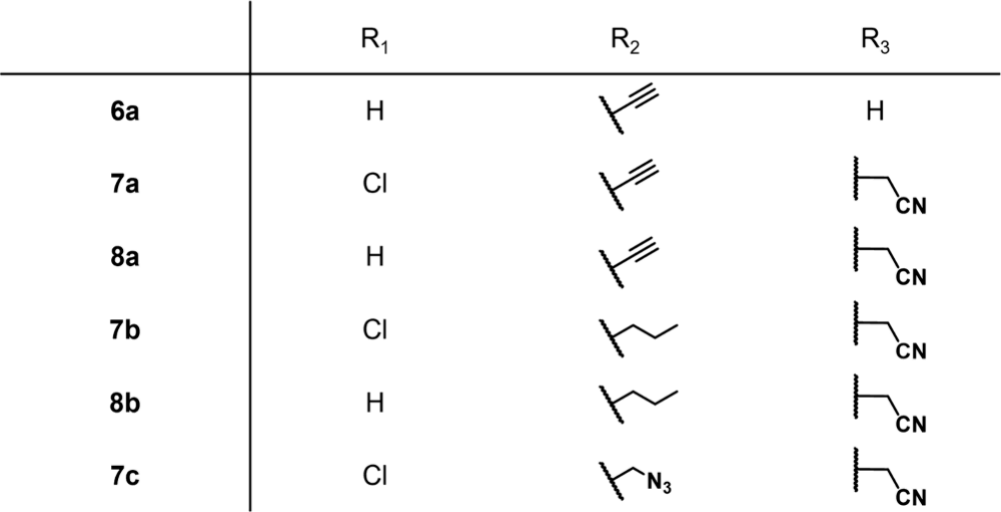
Structure of the OGT Probes^a^

a Compounds **6a**, **7a**, and **8a** are used for fluorescent labeling of the OGT, compounds **7b** and **8b** are used to validate the binding targets of probes for the OGT, and compound **7c** is used for in-cell self-assembly of the OGTAC molecules.

### Covalent Labeling of OGT *In Vitro* and in Cell Lysates with Specificity

Having the probes in hand, we next asked if they covalently labeled purified OGT *in vitro*. To visualize probe-OGT conjugation, we exploited the probes’ terminal alkyne: after allowing the probe to react with the OGT, the alkyne handle was “clicked” to a fluorescent tag (TAMRA-azide) via copper­(I)-catalyzed azide-alkyne cycloaddition (CuAAC) (Figure 2a). In initial tests with purified OGT, compounds **7a** and **8a** (50 μM each, 1 h) robustly labeled the protein, as evidenced by the in-gel fluorescence. The labeling intensity of OGT by **7a** or **8a** was approximately half of that achieved by the pan-reactive positive control, iodoacetamide alkyne (IAA) probe (Figure 2b,c). Labeling by both probes was concentration-dependent (Figure 2d,e) and time-dependent (Figure S1a,b), consistent with covalent bond formation.

**Figure 2:**
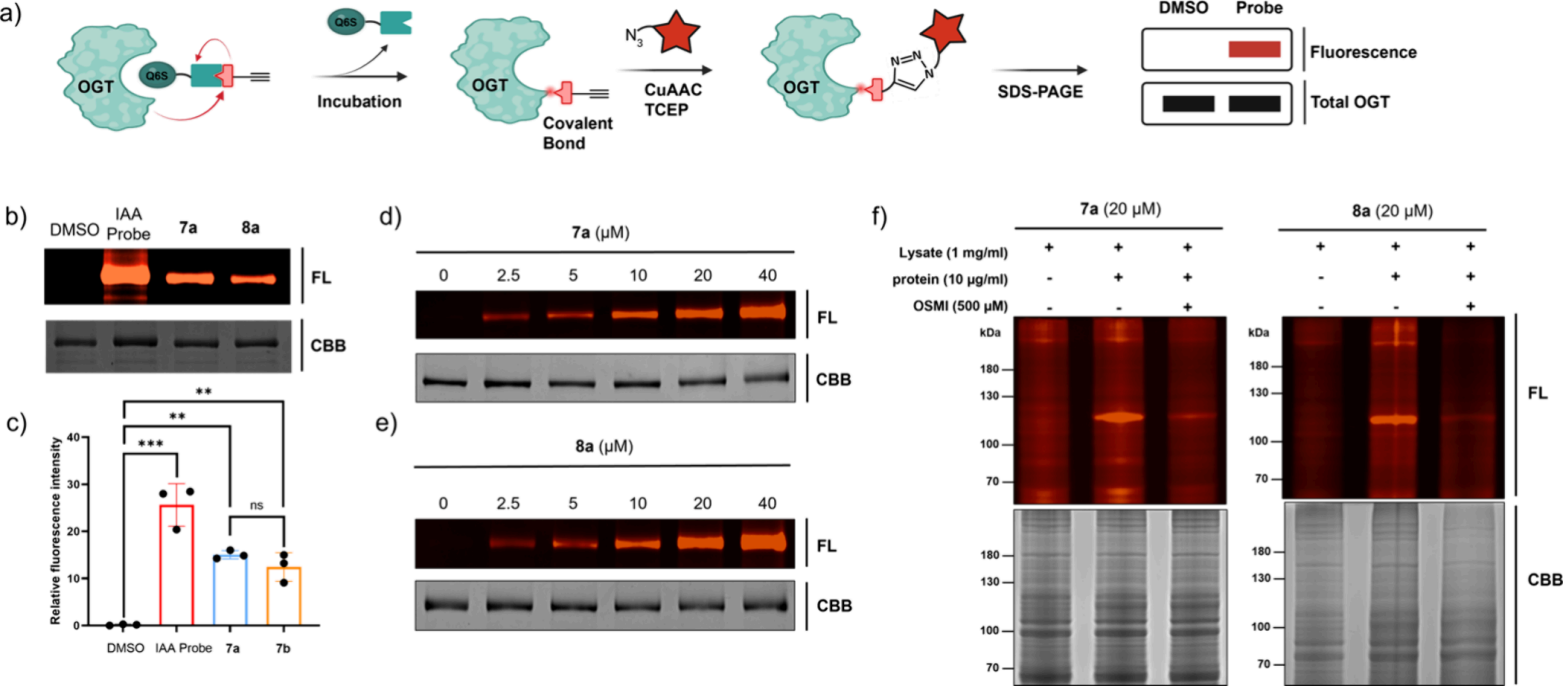
*In vitro* OGT labeling with **7a**, **8a**, and IAA probe. (a) General workflow of recombinant OGT labeling. (b, c) SDS-PAGE and in-gel fluorescence analysis of the OGT labeling. Purified OGT (0.1 μg/μL) was incubated with **7a**, **8a**, or IAA probe (50 μM) in 25 mM HEPES buffer (pH 7.4) at room temperature (r.t.). (d, e) **7a** and **8a** label purified OGT in a dose-dependent manner. (f) **7a** and **8a** label OGT (and potential off-targets) in cell lysate, which could be competed off by the parental compound OSMI-4. All samples were normalized by the BCA assay prior to loading. HEK293T cell lysate with/without purified OGT (10 μg/mL) incubated with **7a** or **8a** (50 μM) for 1 h at r.t. FL, fluorescence signal; CBB, Coomassie Brilliant Blue staining. Error bars represent the mean (SD) from *n* = 3 biologically independent experiments. Statistical significance was assessed using a one-way ANOVA. *p < 0.05; **p < 0.01; ***p < 0.001, ns = no significance. All schematics in this paper were created via BioRender (https://biorender.com).

Control experiments confirmed that labeling required both the warhead and the OGT-directed scaffold. Neither a simple alkyne (propargylamine or heptynoic acid) nor the NASA-lacking analogue **6a** produced significant OGT labeling under the same conditions (Figure S1c,f). Covalent modification was further confirmed by the persistence of strong fluorescent signals on SDS-PAGE gels after extensive washing of the protein samples postclick chemistry (Figure S1c). To further confirm that OGT labeling is dependent on the Q6S scaffold rather than nonspecific modification, we designed and synthesized probe **FR01** and **FR02** (Figure S1d and Scheme S2). **FR02** lacks the Q6S moiety present in **7a** but retains the NASA warhead. The results showed a significant reduction in the efficiency of the OGT labeling with **FR02** compared to **7a** (Figure S1e). Although a faint band was observed, it may be attributed to the inherent high reactivity of the NASA warhead. This confirms that efficient labeling of OGT depends on the Q6S scaffold. Moreover, preincubation with excess OSMI-4 (25×) before probe treatment abrogated OGT labeling by **7a**/**8a** (Figure S1g), indicating that the probes engage OGT at the intended binding site. Together, these results demonstrate that **7a** and **8a** covalently modify the OGT in a target-specific manner via the designed LDR mechanism.

To assess probe selectivity in complex cellular environments, we attempted the labeling of endogenous OGT in cell lysates. Due to the low endogenous abundance of OGT, no distinct band was observed at the expected molecular weight (Figure 2f). However, spiking the lysate with purified OGT (10 μg/mL) prior^43^ to probe incubation resulted in clear labeling of a band at ∼110 kDa, corresponding to OGT, by both **7a** and **8a** (Figure 2f). Even at half of the spiked OGT concentration (5 μg/mL), **7a** labeling remained evident (Figure S1h). Importantly, this labeling was effectively competed by excess OSMI-4, confirming target engagement specificity. Cumulatively, the data confirm that our probes can covalently label OGT with specificity *in vitro* and in complex cellular context.

### Probes Engage OGT while Preserving Its Catalytic Activity

To assess whether the probes retain the catalytic activity of OGT, a prerequisite for constructing OGTACs, we evaluated the effects of compounds **7a** and **8a** on OGT activity both *in vitro* and *in cellulo*. Using the UDP-Glo assay, neither **7a** nor **8a** inhibited purified OGT activity *in vitro* across the concentration range of 1–50 μM (Figure 3a). In HEK293T cells, treatment with probes (up to 100 μM, 6 h) likewise showed no significant reduction in global O-GlcNAcylation (RL2 signal) versus controls, confirming maintained enzymatic activity. Crucially, both probes contrasted sharply with OSMI-4, which abolished activity at 5 μM (Figure 3b). Thus, unlike OSMI-4, our probes engage OGT without inhibiting its catalytic function.

**Figure 3:**
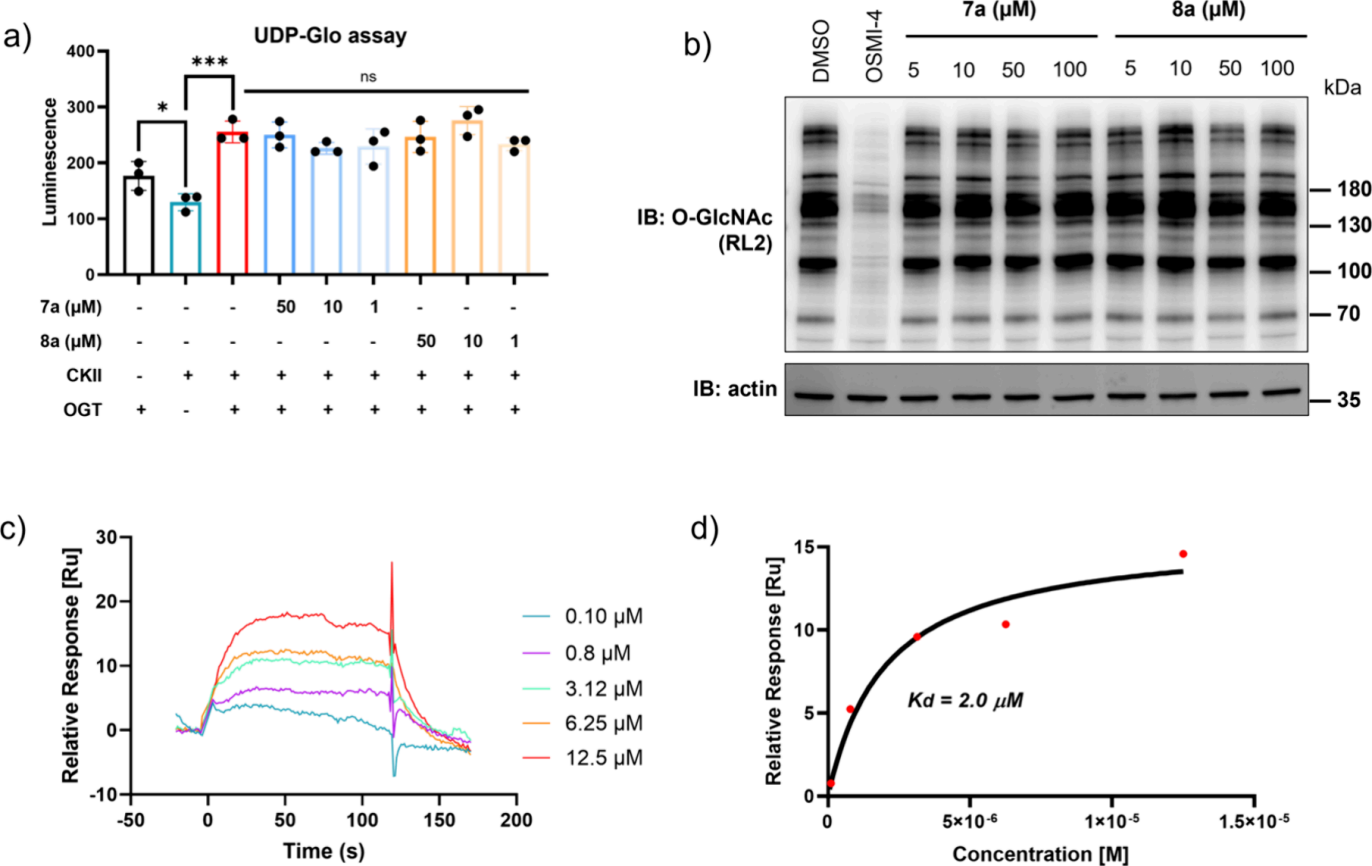
Inhibition and binding affinity of probes for OGT. (a) Probes **7a** and **8a** show no observable inhibition of OGT activity *in vitro*. Activity was measured using a luminescence-based coupled enzyme assay (UDP-Glo; Promega) with 40 μM UDP-GlcNAc and 125 μM CKII peptide substrate. (b) Effect of OSMI-4, **7a**, and **8a** on OGT activity *in cellulo*. Immunoblot analysis of the global O-GlcNAcylation level in lysates from HEK293T cells treated directly with **7a** or **8a** at the indicated concentrations. (c, d) Representative SPR sensorgrams and calculated *K*_d_ for binding of **7a** to immobilized OGT. Affinity curve fitting was performed with the Biacore T200 software using a steady-state affinity model to calculate disassociation constant (*K*_d_). Error bars represent the mean (SD) from *n* = 3 repeating groups. Statistical significance was assessed using a one-way ANOVA. *p < 0.05; **p < 0.01; ***p < 0.001, ns = no significance.

Subsequently, we quantified the dissociation constant (*K*_d_) using surface plasmon resonance (SPR), revealing robust binding of both probes to OGT. **7a** exhibited higher affinity (*K*_d_ = 2.0 μM) than **8a** (*K*_d_ = 28.3 μM) (Figure 3c,d and Figure S2a,b). This affinity results align with established structure–activity relationships (SARs), of which the introduction of a chlorine atom at the ortho position of the sulfonamide moiety in **7a** enhances complementarity within OGT’s binding pocket.^29^ Furthermore, we also validated the binding affinity of probe **8a** using microscale thermophoresis (MST), yielding a *K*_d_ value in close agreement with the SPR measurement (Figure S2c). Together, these results establish that **7a** and **8a** are noninhibitory OGT ligands—they bind to OGT with measurable affinity while preserving the enzyme’s activity—satisfying a key design criterion for OGTACs.

### LC-MS/MS Maps Six Shared Covalent Binding Sites across OGT

Having confirmed that the probes engage OGT via the intended LDR mechanism, we next mapped the covalent attachment sites on OGT. A direct mass-spectrometric analysis of **7a**/**8a**-adducted OGT is complicated by the probes’ terminal alkynes, which can undergo spurious oxidation or be hard to ionize.^44,45^ We therefore synthesized alkyne-free analogs **7b** and **8b** for site-mapping studies. Competitive in-gel labeling assays demonstrated that pretreatment with **7b** or **8b** (50 μM, 30 min) markedly attenuated the fluorescence signal of **7a**- or **8a**-labeled proteins upon CuAAC conjugation (Figure S3), indicating shared binding sites. To pinpoint covalent attachment sites, purified OGT was incubated with **7b** or **8b** (50 μM, 1 h, r.t.), trypsin-digested, and analyzed by LC-MS/MS. Crucially, successful LDR engagement would release the Q6S leaving group while forming a covalent bond between the NASA moiety and lysine residues, yielding a characteristic mass shift of +126.1 Da (Figure 4a). MS analysis revealed high sequence coverage: >70% for all groups (DMSO, **7b**, and **8b**) (Table S1) and robust spectral counts: DMSO control (0 modified peptides), **7b** (18), and **8b** (15) (Table S1). Both probes modified eight lysine residues each, with six overlapping sites (K337, K385, K440, K550, K623, and K717; Figure 4b and Figure S4). Representative MS/MS spectra localized the modification to K440 by **7b** (Figure 4c) and **8b** (Figure 4d), evidenced by b/y-ion series with the +126.1 Da shift. To exclude potential nonspecific binding artifacts arising from high probe concentrations, we reduced the **7b**’s concentration and performed parallel incubations with both 5 μM and 50 μM of **7b**. Subsequent LC-MS/MS analysis revealed significant overlap between the results obtained at both concentrations. Intersection analysis identified residues K337, K385, K440, and K623 as exhibiting high-confidence probe modification even at the lower 5 μM concentration. Corresponding MS2 spectra validating these modifications are presented in Figure S5.

**Figure 4:**
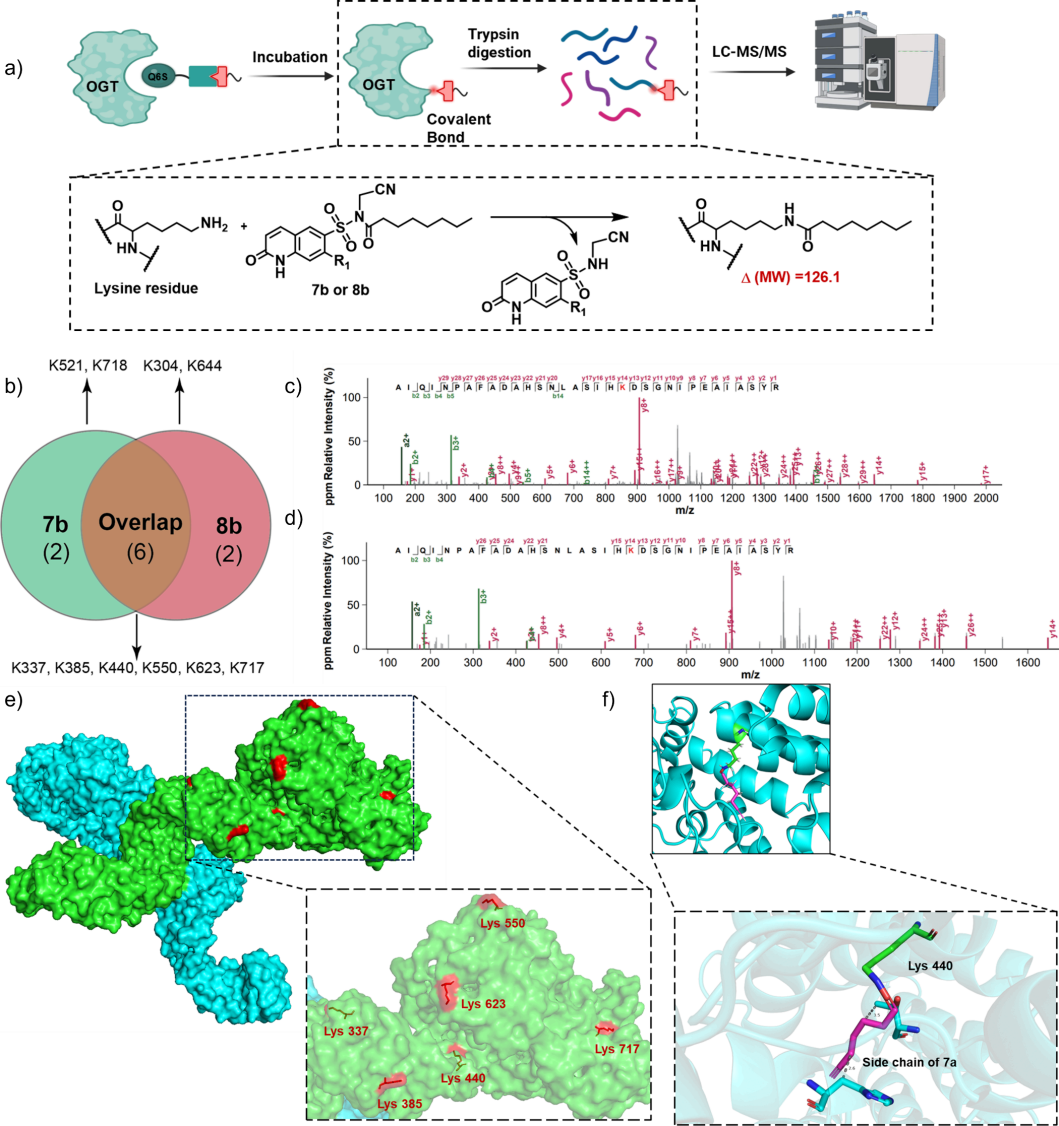
Identification of OGT binding sites for covalent probes. (a) Workflow for binding site mapping. Purified OGT was incubated with **7b** or **8b** (50 μM), trypsin-digested, and subjected to LC-MS/MS analysis. Modified peptides were identified by mass shifts corresponding to probe adducts. (b) Overlap of modified residues. Venn diagram displays eight unique lysine residues modified by **7b** and **8b**, with six sites shared by both probes. (c, d) Site-specific MS/MS spectra. Fragmentation patterns confirm covalent modification of K440 by **7b** (c) and **8b** (d), with diagnostic b/y ions localizing the modification site (red highlight). (e) Distribution of predicted lysine residues on the OGT dimer structure (PDB: 7YEA). The figure was generated using PyMOL (Version 3.0.3). (f) Covalent docking simulation of compound **7a** with OGT K440 (PDB: 4N3C). The simulation depicts the formation of a covalent bond. The lysine residue is shown in green, and the magenta moiety represents the side chain of the molecule left behind after covalent bond formation.

Analysis of shared binding sites between the two probes revealed that the engaged residues K337, K385, K440, and K550 are located within the tetratricopeptide repeat (TPR) domain and the TPR-catalytic domain linker region of OGT. In contrast, K623 and K717 reside within the catalytic domain (which are located in the N-terminal domain and intermediate domain, respectively).^46^ Structural mapping of these amino acid residues using the OGT dimer model (PDB: 7YEA)^47^ revealed that only K440 is located within the binding pocket, while all others are distributed around its periphery (Figure 4e). This spatial distribution of multiple covalent modification sites was unexpected based on our initial assumption that the Q6S moiety could selectively bind the catalytic pocket of OGT. We propose the following explanation for multisite labeling: the Q6S group likely guides the probe to dock near OGT’s active site, but given the probe’s moderate affinity, it may not lock in place long enough to react at a single defined residue. Instead, the probe can diffuse along the solvent exposed surface of OGT, reacting with any suitably positioned lysine before dissociating. In support of this model, covalent docking simulations for K440 (the lysine in binding pocket) showed a binding pose and covalent bond formation with **7a** (Figure 4f), confirming that at least one of the identified sites is compatible with a direct active-site engagement. In summary, the LDR probes label multiple lysine residues on OGT (a likely consequence of their moderate binding affinity), including the expected lysine residue in the ligand binding pocket.

### Self-Assembled OGTACs Achieve Targeted O-GlcNAcylation without Perturbing Global O-GlcNAcylation Levels

Based on the preceding experimental findings, we have successfully identified a noninhibitory covalent ligand capable of selectively targeting lysine residues on OGT. Capitalizing on this discovery, we next proceed to develop bifunctional OGTACs. Initially, we synthesized and tested bifunctional OGTACs by directly linking the modified OGT ligand to the BRD4 ligand JQ1. However, no proximity-induced ternary complex formation or detectable increase in BRD4 O-GlcNAcylation was observed (data not shown). This failure may be attributed to (1) the high molecule weight of bifunctional molecule, which may diminish the cell permeability,^48^ and (2) a potential mismatch in binding affinities between the two ligands and their respective protein targets^49^—notably, the *K*_d_ of JQ1 for BRD4 ^50^ is several orders of magnitude higher than that of compound **7a** for OGT. Thus, we employed an in-cell self-assembly strategy as a remedy (Figure 5a). In this approach, we used the azide ligand **7c** instead of the alkyne ligand **7a** to label OGT. This strategic modification enabled the utilization of copper-free click chemistry for assembling the bifunctional OGTAC molecule *in cellulo*, thereby (1) improving overall cell permeability, (2) addressing the potential mismatch in binding affinities between the two ligands, and (3) effectively circumventing the cytotoxicity associated with copper ions.

**Figure 5:**
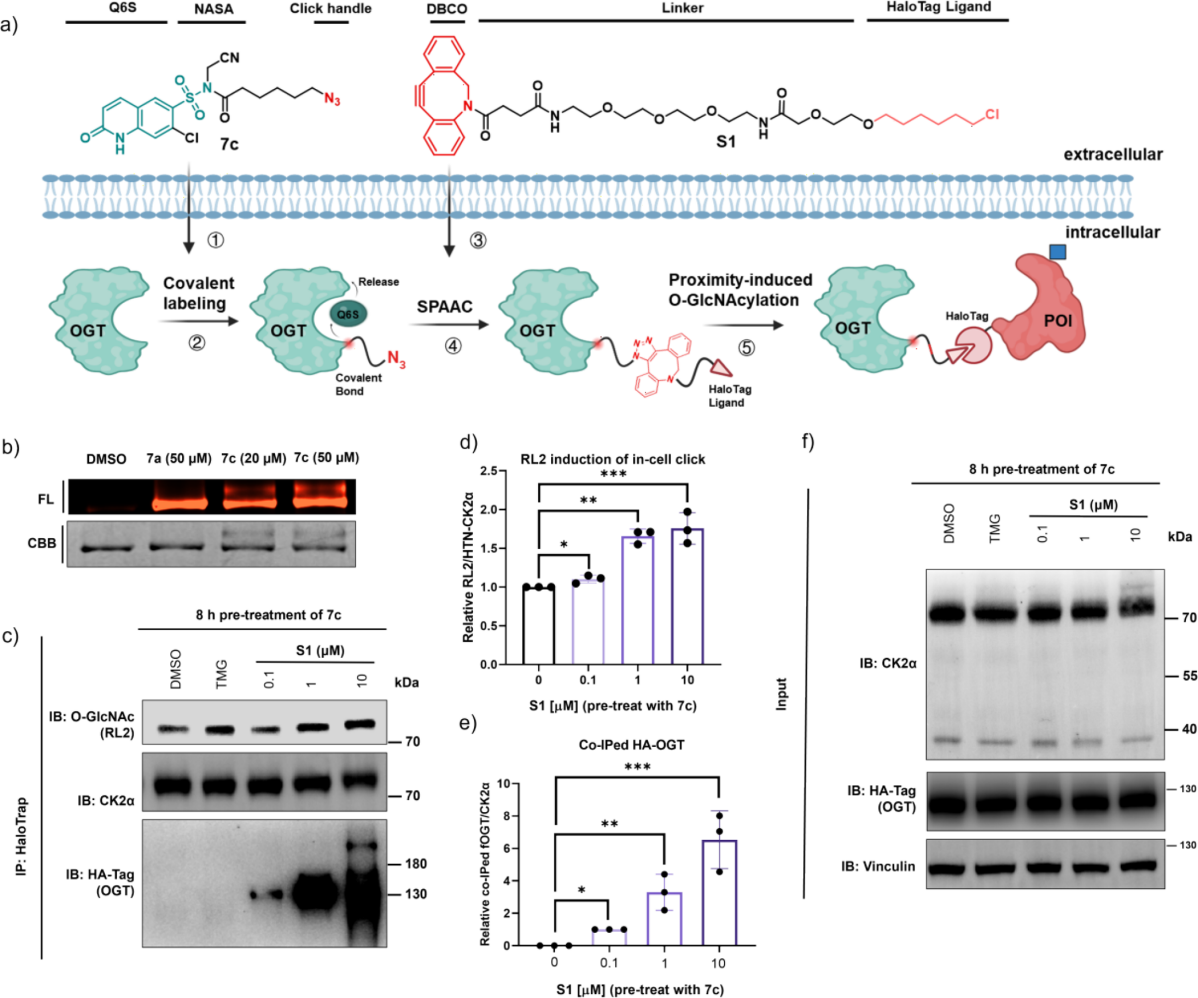
Design and development of self-assembled OGTAC. (a) Schematic diagram of the experimental workflow for in-cell self-assembly of OGTACs. The numbered circles (1–5) indicate the key experimental steps. (b) *In vitro* OGT labeling efficiency analysis of **7a** and **7c**. (c) OGTACs specifically elevate the O-GlcNAcylation level of HTN-CK2α through proximity induction. (d) Quantification of relative HTN-CK2α O-GlcNAcylation (RL2 signal normalized to CK2α IP) fold changes over DMSO from the panel. (e) Quantification of relative co-IP OGT (HA signal normalized to total CK2α IP) compared to the 0.1 μM S1 treatment group from the panel. (f) Expression level of HTN-CK2α and HA-OGT. HEK293T cells cotransfected with HTN-CK2α and HA-OGT were treated with probe **7c** (200 μM) for 8 h. Following medium removal and replacement, cells were incubated with varying concentrations of probe S1 for 16 h. Cells were then harvested, and target protein O-GlcNAcylation levels were analyzed by Western blotting. Error bars represent the mean (SD) from *n* = 3 biologically independent experiments. Statistical significance was assessed using one-way ANOVA. *p < 0.05; **p < 0.01; ***p < 0.001.

*In vitro* labeling assays demonstrated that **7c** maintained comparable labeling efficiency toward OGT to that of **7a** (Figure 5b). Initial validation of the live-cell OGT labeling confirmed that probe **7c** effectively labeled OGT at a concentration of 200 μM and demonstrated moderate selectivity (Figure S6a). Furthermore, CCK-8 assays indicated no significant cytotoxicity for **7c** in HEK293T cells (IC_50_ > 100 μM, Figure S6b).

We then evaluated the OGTAC system using coimmunoprecipitation (co-IP) assays through a dual-transfection approach. Cells coexpressing HaloTag-N-terminal-CK2α (HTN-CK2α) and HA-OGT were pretreated with probe **7c** (200 μM, 8 h) to covalently label OGT. Following medium removal and replacement, varying concentrations of the DBCO-PEG3-HaloTag ligand (probe **S1**, Figure 5a and Scheme S3) were added to facilitate in-cell OGTAC self-assembly via copper-free strain-promoted azide-alkyne cycloaddition (SPAAC). After 16 h of incubation, cells were harvested. The results revealed an increase in HTN-CK2α O-GlcNAcylation at an **S1** concentration of 10 μM compared to the DMSO control (Figure 5c,d,f). Furthermore, co-IP demonstrated the dose-dependent formation of the ternary complex, evidenced by the interaction between HTN-CK2α and HA-OGT (Figure 5c,e,f). To confirm that the O-GlcNAcylation occurs specifically at the native S347 site of CK2α, we performed a control experiment. We transfected cells with HTN-CK2α and HTN-CK2α^S347A^ and treated them under identical conditions with probe **7c** (200 μM) followed by **S1** (10 μM), as described previously. The results demonstrated that O-GlcNAcylation of CK2α was not observed in the S347A mutant group, indicating that the modification is specific to the native S347 site (Figure S7). Furthermore, in contrast to the broad increase in global RL2 levels induced by TMG, our probe treatment did not cause a significant upregulation of global O-GlcNAcylation (Figure S7). Additionally, the alkyne-terminated probe **7a** failed to self-assemble in cells and therefore did not induce proximity or alter O-GlcNAcylation, indicating that a terminal azide group is critical for this self-assembly strategy (Figure S8). Collectively, these data establish proof-of-concept for self-assembling OGTAC as a novel strategy to achieve targeted protein O-GlcNAcylation.

## Discussion

Despite recent advances in chemical biology tool development, dissecting the site-specific functional consequences of O-GlcNAcylation remains a formidable challenge. The emergence of proximity-inducing OGTAC molecules offers a promising path forward. However, while numerous ligands have been developed to target OGT, the majority act as inhibitors, which precludes their use in constructing noninhibitory OGTACs necessary for functional studies.^51^ Thus, the development of noninhibitory OGT ligands is very important for advancing next-generation OGTAC technologies.

In this work, we addressed this critical gap through an LDR strategy, strategically incorporating a NASA warhead into the OSMI-4 scaffold. This approach yielded covalent probes that effectively labeled OGT *in vitro* and in living cells while preserving enzymatic activity—a prerequisite for targeted O-GlcNAcylation applications. Leveraging this ligand, we established a proof-of-concept for in-cell OGTAC self-assembly. This in-cell assembly strategy effectively circumvents the challenge of poor membrane permeability associated with the large molecular weight (MW) of bifunctional molecules, thus avoiding potential impairment of the ternary complex formation efficiency. Furthermore, it mitigates the risk of failed ternary complex assembly due to a mismatch in the binding affinities of the two constituent ligands for their respective protein targets. In our design, the ligand of HaloTag (**S1**’s chloroalkane chain) exhibits significantly higher affinity for HaloTag than **7c** does for OGT. Therefore, we first pretreated **7c** to ensure extensive labeling of OGT, followed by the addition of **S1**. The highly efficient SPAAC reaction then occurred in the cell, generating the desired bifunctional OGTAC molecule *in situ*. The system successfully induced proximity between OGT and a POI (CK2α), leading to selective enhancement of target protein O-GlcNAcylation without perturbing global cellular O-GlcNAcylation. This validates the core premise of OGTACs as a programmable platform for controlling O-GlcNAcylation signaling.

Unlike strategies that rely on genetic fusion or macromolecular scaffolds, our approach bypasses the need for protein engineering and leverages the inherent cell permeability and chemical availability of OGT ligands. Compared to OGTAC-1,^25^ this new approach can engage fusion-free OGT and also elevate the O-GlcNAcylation levels of specific proteins. This broader functionality makes the system suitable for a wide range of experimental scenarios.

Despite this advance, there are key challenges to address in the future. First, the current lead probe has suboptimal binding affinity and selectivity, to the extent that an OGT overexpression and high concentration of the probe are needed to ensure robust labeling. This limitation likely arises from the scaffold simplification required to install the warhead—in stripping OSMI-4 down to its Q6S core, where some favorable protein-ligand contacts were lost, compromising binding strength. Second, the inherently high electrophilicity of the NASA warhead may contribute to off-target reactions, as suggested by the multiple lysine sites labeled on the OGT and potential background labeling in cells. Consequently, the probe described herein may engage in multitarget labeling events, which precludes its direct use for drawing precise functional conclusions about specific endogenous targets. These issues highlight the trade-offs inherent in our current design. Comprehensive global proteomics studies to rigorously define the off-target profile of this probe and other related OSMI derivatives would be of significant value for the field. Our future work will include designing next-generation probes with enhanced selectivity, the evaluation of which will include detailed proteomic assessments to fully characterize their utility as biological tools.

We plan to enhance the binding affinity of noninhibitory OGT ligands to facilitate their application in non-transfected systems such as targeting the endogenous OGT without the need of overexpression. Achieving high-affinity ligands would enable the design of molecules that more accurately reflect intracellular reality, thereby providing deeper insights into OGT-related biological mechanisms. Such advanced ligands would also exhibit greater potential for drug development, opening avenues for creating OGT-targeted small-molecule therapeutics. To this end, comprehensive studies of the SARs of existing probes are essential to guide the rational design of more effective OGT ligands. We will investigate the amino acid environment within the OGT binding pocket to select the appropriate nucleophilic residue for covalent targeting. Our LC-MS/MS experiments revealed that multiple lysine residues on the OGT are labeled by the probes. A systematic site-directed mutagenesis study would help to pinpoint the major residue(s) undergoing modification, providing a stronger foundation for the rational design of next-generation covalent OGTACs. Beyond the lysine-targeting NASA probes described here, other warheads directed toward different amino acids could be explored. Systematic conjugation of various covalent warheads with the OGT-binding ligands will facilitate the screening of optimal probes. These investigations may also offer strategic guidance for selecting which amino acids—and at which positions—on the OGT should be targeted in the development of bifunctional molecules.

In summary, the identification of a noninhibitory OGT ligand is a crucial step toward precision control of protein O-GlcNAcylation and related PTM editing. While our current probes have several limitations, the LDR-based approach provides a versatile foundation for iterative improvement. Ongoing work will focus on boosting ligand affinity, sharpening selectivity, and improving the efficacy of targeted O-GlcNAcylation induction. Success in these endeavors will pave the way for deploying OGTACs in native biological contexts and for developing drug-like modulators of O-GlcNAc signaling—a transformative capability not yet accessible in the field.

## Supplementary Material



## Notes

Detailed experimental procedures, compound characterization, and supplementary data (e.g., time-dependent and specific labeling of OGT by probes **7a** and **8a**, binding curve of probe **8a** to OGT measured by SPR and MST, etc.) (PDF)
