# Supplementary material for: Ligand-Directed Self-Assembling Chimeras for Targeted Protein O‑GlcNAcylation
Source: ACS Chem Biol. 2025 Dec 5;20(12):2907–16. doi: 10.1021/acschembio.5c00684 (PMC12723682; doi:10.1021/acschembio.5c00684)
Supplement: Supplementary file 1 [file cb5c00684_si_001.pdf]

**Ligand-Directed Self-Assembling Chimeras for Targeted Protein O-GlcNAcylation**

**Supporting Information**

Zhihao Guo,[1,#] Tongyang Xu,[1,#] Khadija Shahed Khan,[1,2,#] Stephan Scheeff,[1] Yao Qin,[1] Sin-Yi Yu,[1] Richard Lo,[1] Yuanpei Li,[1] Yalun Xie,[1] Bowen Ma,[1] Yunpeng Huang,[1] Hillary Yui-Yan Yip,[3] Clive Yik-Sham Chung,[3] Tomonori Tamura,[4] Itaru Hamachi,[4] and Billy Wai-Lung Ng*,[1,5,6,7]

1. Guangdong-Hong Kong-Macao Joint Laboratory for New Drug Screening, School of Pharmacy, The Chinese University of Hong Kong, Sha Tin, Hong Kong

2. School of Biomedical Sciences, Faculty of Medicine, The Chinese University of Hong Kong, Sha Tin, Hong Kong

3. School of Biomedical Sciences, Li Ka Shing Faculty of Medicine, The University of Hong Kong, Pok Fu Lam, Hong Kong

4. Department of Synthetic Chemistry and Biological Chemistry, Graduate School of Engineering, Kyoto University, Katsura, Kyoto 615-8510, Japan.

5. Li Ka Shing Institute of Health Sciences, Faculty of Medicine, The Chinese University of Hong Kong, Sha Tin, Hong Kong

6. Gerald Choa Neuroscience Institute, The Chinese University of Hong Kong, Sha Tin, Hong Kong

7. Peter Hung Pain Research Institute, Faculty of Medicine, The Chinese University of Hong Kong, Sha Tin, Hong Kong

#: Authors contributed equally to this paper.

*: billyng@cuhk.edu.hk

## Supporting Figures, Tables and Schemes

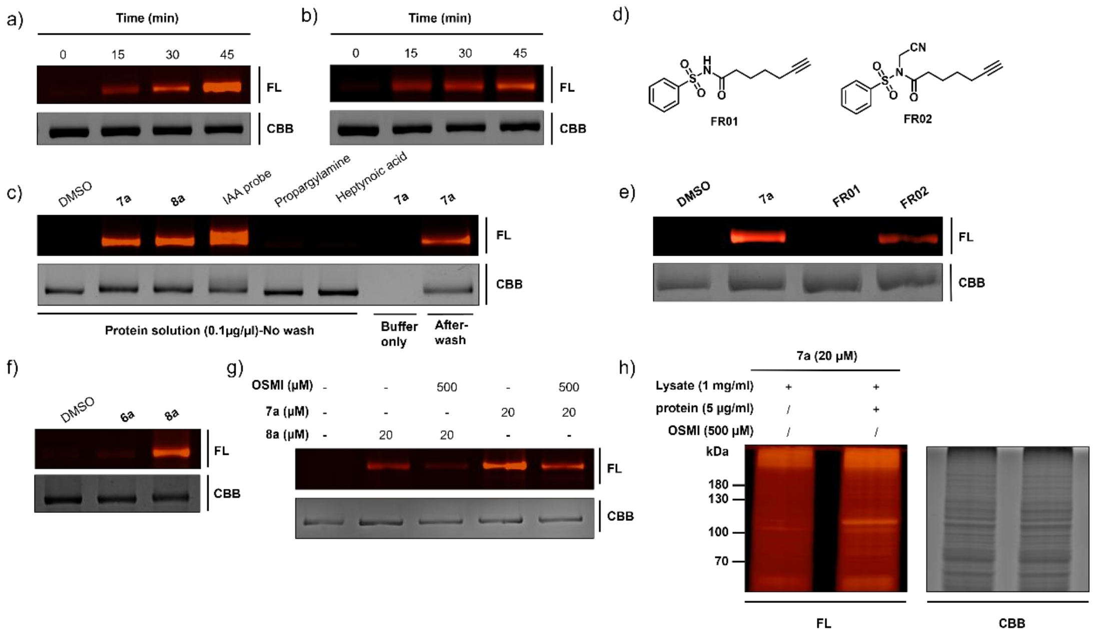


**Figure S1**. Time-dependent and specific labeling of OGT by probes **7a** and **8a**. (a-b) Time-dependent labeling of OGT by **7a** and **8a**. (c) Specific labeling of OGT by **7a** and **8a**, with no significant nonspecific labeling observed by alkyne controls (propargylamine and heptynoic acid). d) Chemical structures of probes **FR01** and **FR02**. e) Comparison of OGT labeling efficiency between probe **7a and FR02**, each at 20 μM. **FR02** exhibits a significant reduction in labeling capability. (f) The probe **6a**, lacking the NASA group, failed to achieve covalent labeling of OGT under identical conditions. (g) Competition with OSMI-4 attenuates probe labeling. (h) Detectable labeling of OGT by **7a** in HEK293T cell lysate spiked with recombinant OGT (5 μg/ml).

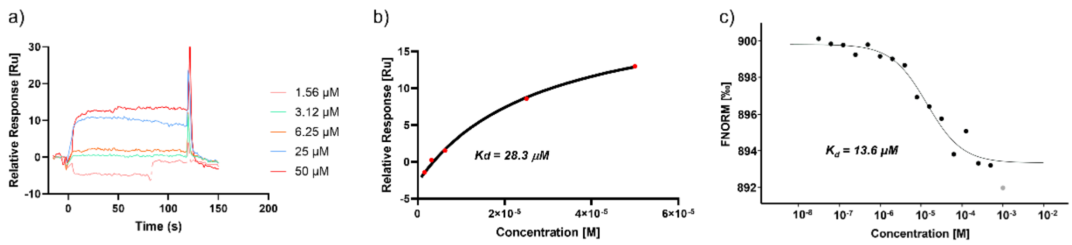


**Figure S2**. Binding curve of probe **8a** to OGT measured by SPR and MST. Affinity curve fitting was performed with the Biacore T200 software using a steady-state affinity model to

calculate disassociation constant ($K_d$). MST curve fitting and $K_d$ determination were performed in MO.Affinity Analysis software.

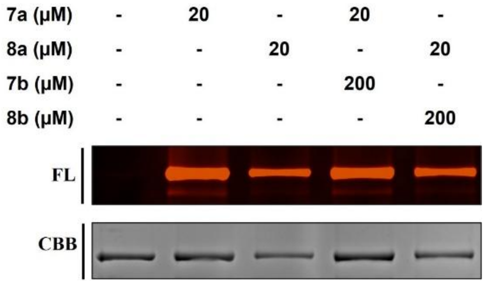


**Figure S3**. Competitive inhibition of probe labeling. Recombinant OGT were pretreated with or without compounds **7b** or **8b** (50 μM) for 30 min, followed by incubation with fluorescent probes **7a** or **8a** (10 μM). Subsequent CuAAC-mediated conjugation to a TMARA-azide tag revealed that pretreatment with **7b**/**8b** attenuated the in-gel fluorescence signal of **7a**/**8a**-labeled proteins, indicating competitive binding at shared sites.

| Code | **Sequence Coverage[1]** | **Modified Peptides[2]** | **Spectral Counts[3]** |
|---|---|---|---|
| **DMSO** | 71.8% | 0 | 0 |
| **7b** | 75.1% | 8 | 18 |
| **8b** | 86.9% | 8 | 15 |

**Table S1**. MS-based identification of OGT covalent modification sites

[1] Sequence coverage: Percentage of OGT amino acid sequence detected by tryptic peptides across all groups. Coverage >70% ensures comprehensive detection of potential modification sites, calculated via pFind against His-OGT.

[2] Modified peptides: Unique peptide-spectrum matches (PSMs) with +126.1 Da mass shift localized to lysine residues. DMSO control values confirm absence of non-specific modifications.

[3] Spectral counts: Total MS/MS events identifying modified peptides.

1 MASSVGNVADSTEPTKRMLSFQGLAELAHREYQAGDFEAAERHCMQLWRQEPDNTGVLLLLSSIHFQCRRLDRSAHFSTLAIKQNPLLAEAYSNLGNVYKERGQLQEAIE
111 HYRHALRLKPDFIDGYINLAAALVAAGDMEGAVQAYVSALQYNPDLYCVRSDLGNLLKALGRLEEAKACYLKAIETQPNFAVAWSNLGCVFNAQGEIWLAIHHFEKAVTLDP
223 NFLDAYINLGNVLKEARIFDRAVAAYLRALSLSPNHAVVHGNLACVYYEQGLIDLAIDTYRRAIELQPHFPDAYCNLANALKEKGSVAEAEDCYNTALRLCPTHADSLNNLANI
337 KREQGNIEEAVRLYRKALEVFPEFAAAHSNLASVLQQQGKLQEALMHYKEAIRISPTFADAYSNMGNTLKEMQDVQGALQCYTRAIQINPAFADAHSNLASIHKDSGNIPEA
449 IASYRTALKLKPDFPDAYCNLAHCLQIVCDWTDYDERMKKLVSIVADQLEKNRLPSVHPHHSMLYPLSHGFRKAIAERHGNLCLDKINVLHKPPYEHPKDLKLSDGRLRVGY
561 VSSDFGNHPTSHLMQSIPGMHNPDKFEVFCYALSPDDGTNFRVKVMAEANHFIDLSQIPCNGKAADRIHQDGIHILVNMNGYTKGARNELFALRPAPIQAMWLGYPGTSG
671 ALFMDYIITDQETSPAEVAEQYSEKLAYMPHTFFIGDHANMFPHLKKKAVIDFKSNGHIYDNRIVLNGIDLKAFLDSLPDVKIVKMKCPDGGDNADSSNTALNMPVIPMNTIAE
785 AVIEMINRGQIQITINGFSISNGLATTQINNKAATGEEVPRTIIVTTRSQYGLPEDAIVYCNFNQLYKIDPSTLQMWANILKRVPNSVLWLLRFPAVGEPNIQQYAQNMGLPQNR
900 IIFSPVAPKEEHVRRGQLADVCLDTPLCNGHTTGMDVLWAGTPMVTMPGETLASRVAASQLTCLGCLELIAKNRQEYEDIAVKLGTDLEYLKKVRGKVWKQRISSPLFNTK
1011 QYTMELERLYLQMWEHYAAGNKPDHMIKPVEVTESA

**7b** **7b & 8b** **8b** **Active site**

**Figure S4**. Comprehensive mapping of covalent modification sites on OGT. The full-length OGT sequence is depicted with catalytic residues highlighted in yellow. Sites exclusively modified by **7b** are shown in green, those unique to **8b** in blue, and shared modification sites in red.

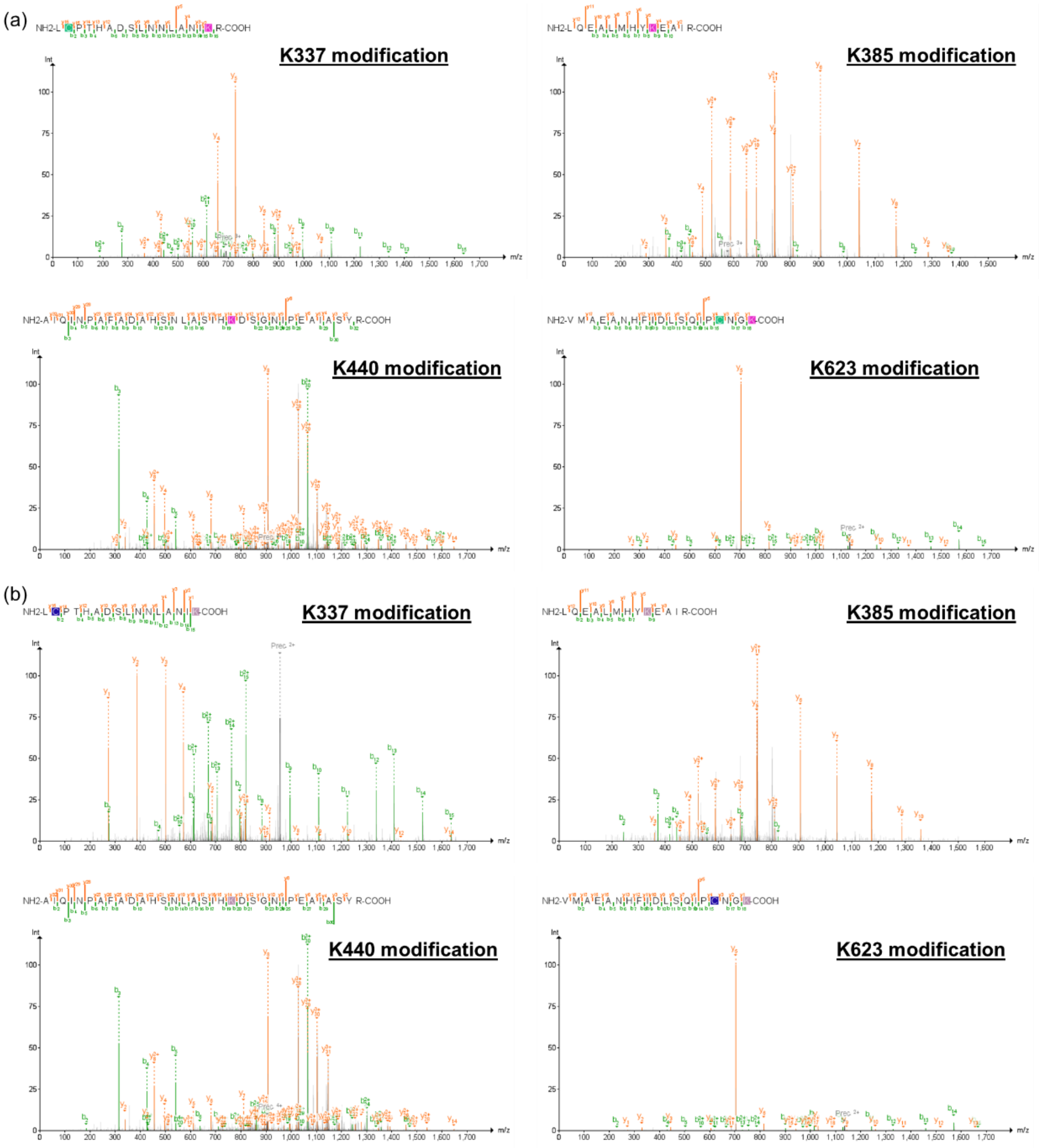

**Figure S5.** MS/MS spectra showing the covalent modification of OGT by **7b** at (a) 5 μM and (b) 50 μM. K337, K385, K440 and K623 are the sites of modification with the highest spectral counts in the experiment.

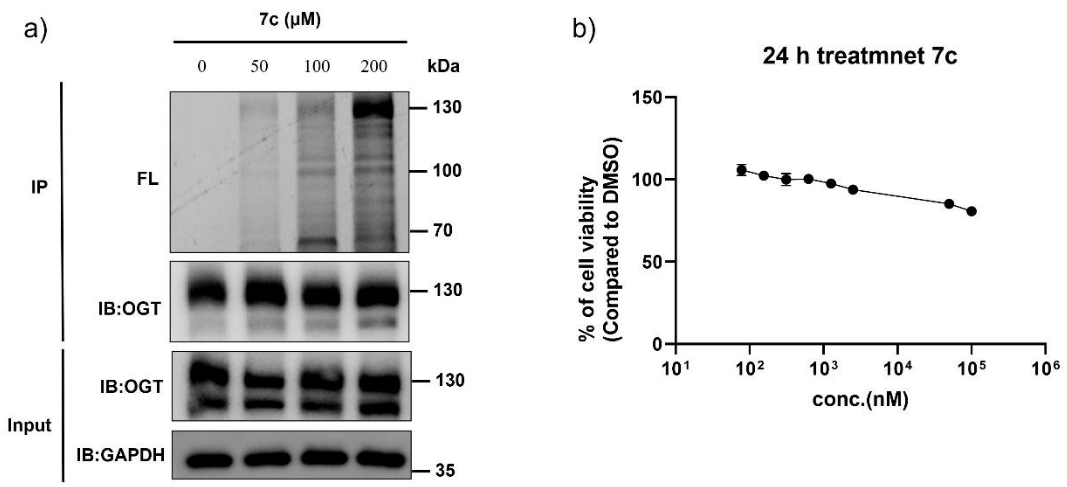


**Figure S6.** a) Labeling efficiency of probe **7c** for OGT protein in live-cells. HEK293T cells over expressing HA-OGT were incubated with varying concentrations of **7c** for 4 h. Following cell lysis, the target protein was enriched using anti-HA affinity beads. TAMRA fluorophore was subsequently conjugated to probe-labeled OGT via click reaction. Labeling efficiency was visualized by in-gel fluorescence detection via SDS-PAGE, and total OGT levels were verified by western blotting. b) Cytotoxicity evaluation of compound **7c** in HEK293T cells. Cells were treated with **7c** at different concentrations for 24 h, and cell viability was measured using the CCK-8 assay.

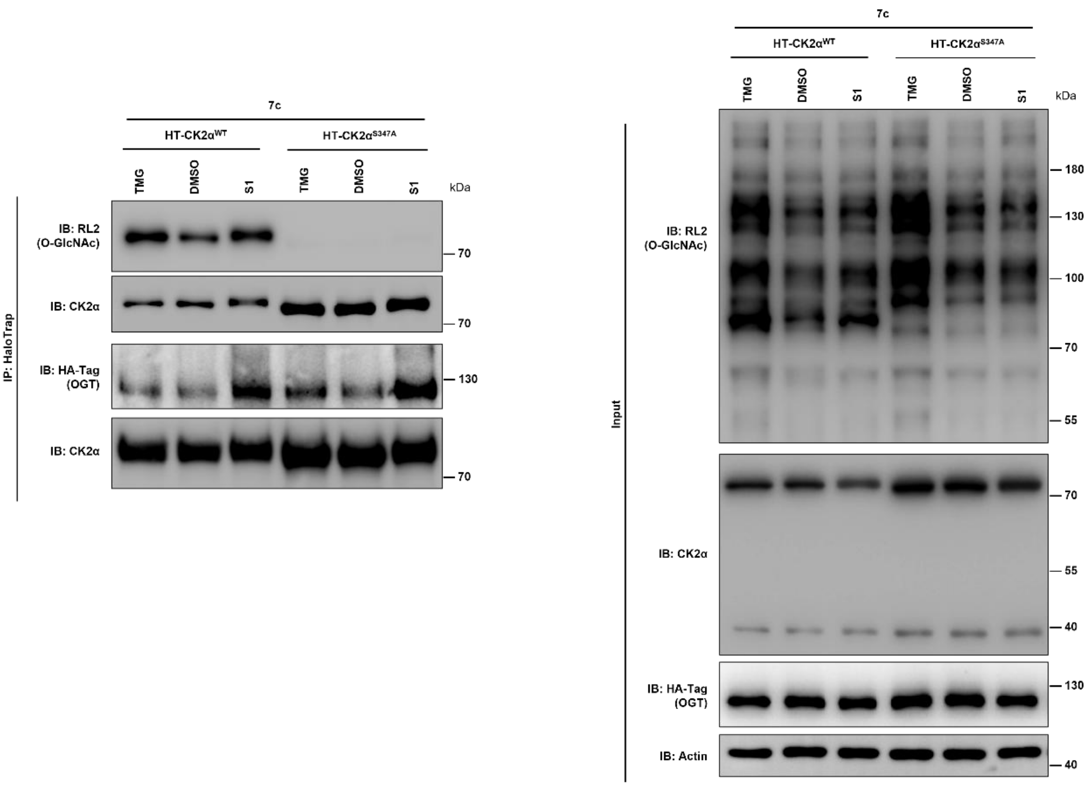


**Figure S7**. Probe **7c**/**S1** assembly specifically induces O-GlcNAcylation at S347 of CK2α and does not elevate global O-GlcNAc levels. Western blot analysis of O-GlcNAcylation on wild-type (WT) and S347A mutant HTN-CK2α. Treatment with the **7c**/**S1** (10 μM) assembly significantly increased O-GlcNAcylation of WT HTN-CK2α, but not the S347A mutant. TMG served as a positive control and similarly showed no modification of the S347A mutant, confirming S347 as the modification site. Global O-GlcNAc levels were assessed using the RL2 antibody. Unlike TMG treatment, which caused a pronounced increase in global O-GlcNAcylation, the **7c**/**S1** assembly did not alter overall RL2 signal, demonstrating its site-specific action.

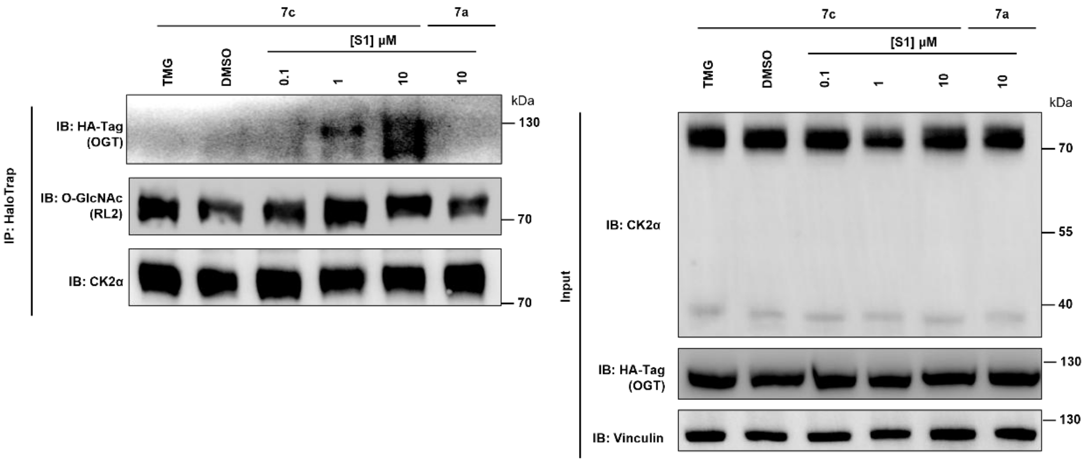


**Figure S8.** Failure of OGTAC self-assembly with alkyne-terminated compound, **7a**, which is a negative control. While OGT labeled with **7c** enabled **S1**-induced intracellular OGTAC self-assembly, assembly failed with the alkyne-terminated compound **7a**.

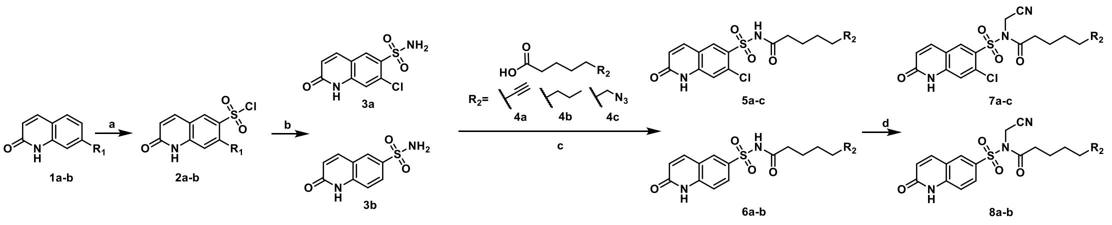


**Scheme S1**. Four-step synthesis yields a family of quinolinone-6-sulfonamide derivatives for functionalization. a) $HSO_3Cl$, 148°C, 3 h. b) Ammonia water (25%), reflux, 3 h. c) EDCI, DMAP, DIPEA, r.t., 72 h. d) Iodoacetonitrile, DIPEA, r.t., 48 h.

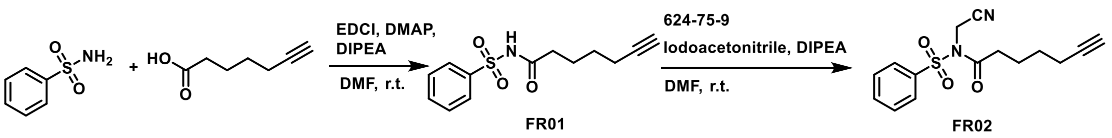


**Scheme S2.** The synthesis route of control probe **FR02**

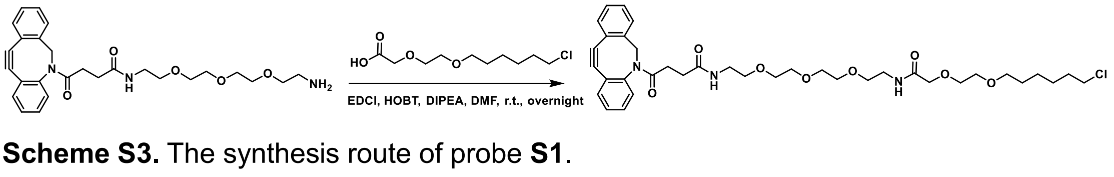


**Scheme S3.** The synthesis route of probe **S1**.

## Method and Material

**Cell culture, DNA Constructs and Reagents**

Experiments were performed using the human embryonic kidney 293T cells HEK293T (kind gifts from Prof. Alfred Sze-Lok Cheng, CUHK). Cells were cultured and maintained at 37°C with 5% CO2 in Dulbecco´s modified Eagle´s medium (DMEM, Gibco-Invitrogen) supplemented with 10% fetal bovine serum (Gibco-Invitrogen) and 1% penicillin and streptomycin.

For plasmids, FKBP12$^{F36V}$-HA-OGT (HA-OGT), pHaloTag vector, and HaloTag-CK2α were obtained as previously described.[1]

Transient transfection was conducted according to the manufacturer’s protocol using Lipo2000 transfection reagent (AboRo, RL0401).

**Protein Purification**[2]

Following expression of human ncOGT (full length, 1–1046) in E. coli, cells were resuspended in a buffer containing 25 mM imidazole, 10 % glycerol, 250 mM NaCl, and 25 mM HEPES, pH 7.5, 5 mM DTT. Protease inhibitor tablet (Roche) was also added to the lysis buffer. Following lysis by sonication and French press, the protein was purified by nickel affinity chromatography and eluted in the same buffer with 250 mM instead of 25 mM imidazole. Fractions containing pure protein were then dialyzed in 25 mM HEPES, pH 7.5, 40 mM NaCl, 0.5 mM EDTA, and 5 mM DTT. Store the protein at -80°C refrigerator.

**Protein Labeling**

For purified OGT, both affinity labeling and click reactions were performed on ice, unless indicated otherwise. The protein was diluted to a concentration of 0.1 mg/mL in assay buffer (25 mM HEPES, pH 7.5, 250 mM NaCl, 10% glycerol (v/v)). Subsequently, 38 μL aliquots of the diluted OGT were incubated for 1 h at room temperature with 2 μL of probe at the indicated concentrations or vehicle control (1% DMSO (v/v)). The click reaction was initiated by adding 3.3 μL of click-mix to the reaction mixture. The click-mix was prepared immediately before use by vigorous mixing of the following components: 1) 1 mM $CuSO_4$

(0.8 μL/reaction, from a 50 mM aqueous stock solution); 2)100 μM TBTA (2 μL/reaction, from a freshly prepared 2 mM stock solution in 1:4 DMSO:t-butanol); 3) 1 mM TCEP (0.4 μL/reaction, from a freshly prepared 100 mM aqueous stock solution); 4) 25 μM TAMRA-Azide (MCE, HY-151857) or TAMRA alkyne (Vectorlabs, CCT-TA108) (0.1 μL/reaction, from a 10 mM stock solution in DMSO). Following a 1 h incubation at room temperature (r.t.), the reactions were quenched by adding 15 μL of 4× SDS loading buffer. Proteins were then resolved by sodium dodecyl sulfate polyacrylamide gel electrophoresis (SDS-PAGE) using 8% polyacrylamide gels (gels made using One-Step PAGE Gel Fast Preparation Kit (8%, Vazyme, E302-01)). In-gel fluorescence was detected using the Rhodamine channel (Bio-Rad). Subsequently, to visualize total protein as a loading control, gels were stained for 15 min with Coomassie Blue Fast Staining Solution (without decolorization; Vazyme) and scanned using the Coomassie Blue channel (Bio-Rad) on the imager.

**Competitive Labeling Assays in Purified OGT.**

Prior to the labeling experiment, purified OGT was diluted to a concentration of 0.1 mg/mL in assay buffer and incubated with 25 (OSMI-4) or 5 (**7b** or **8b**) fold concentration competitor or vehicle control (1% DMSO) for 1h on ice, followed by labeling with 20 μM probe (**7a** or **8a**) for 1 h on ice. Samples were then subjected to the click chemistry procedure using the protocol described above.

**UDP-Glo Assay**

The UDP-Glo assay was performed using a UDP-Glo Glycosyltransferase Assay kit (Promega), as described previously.[3] Assays were performed in white 96-well plates. This assay was run with 125 μM CKII peptide acceptor. Reaction volumes were 20 (normal volume plates) μL. Reactions contained the following components: 300 nM His-OGT, 125 μM CKII peptide and 40 μM UDP-GlcNAc in 1x HEPES pH 7.5 supplemented with 12.5 mM $MgCl_2$ and 1 mM DTT. Reactions were incubated for one hour at 25°C and quenched by the addition of an equal volume of UDP-Glo nucleotide detection reagent, prepared and used according to manufacturer guidelines. The quenched reactions were then mixed

briefly by shaking at 100 rpm for 30 s, and incubated for 30 min at r.t. (in dark) and then detected by microplate reader (CLARIOstar® Plus, BMG Labtech) every 10 min for 2 h. Data were analyzed by Microsoft Excel and GraphPad Prism (version 8.0.2). Each sample in this case was run in triplicate, unless otherwise specified.

**OGT Labeling in Cell Lysate**

HEK293T cells (5 × $10^6$ cells) were seeded into 10 cm dishes and cultured for 24 h at 37°C in a humidified 5% $CO_2$ incubator. Cells were harvested and lysed using ice-cold lysis buffer (25 mM HEPES pH 7.5, 250 mM NaCl, 10% (v/v) glycerol, supplemented with protease inhibitor cocktail (MedChemExpress, HY-K0010)), adding 500 µL buffer per dish. Lysates were subjected to three freeze-thaw cycles using liquid nitrogen, followed by centrifugation at 13,500 rpm for 15 min at 4°C. The supernatant was collected, and protein concentration was determined by Bicinchoninic acid (BCA; ThermoFisher, 23225) assay and adjusted to 1 mg/mL. The lysate was aliquoted equally into 1.5 mL microcentrifuge tubes (250 µL per tube). Purified recombinant OGT protein (4 µL of 1.2 µg/µL stock) was added or omitted to achieve final concentrations of 5 or 10 µg/mL in the lysate samples. Prior to probe labeling, samples were incubated on ice for 1 h in the presence or absence of 500 µM OSMI-4. Subsequently, the labeling reaction was initiated by adding either probe **7a** or **8a** to a final concentration of 20 µM, followed by incubation on ice for an additional hour. Click chemistry conjugation was then performed as above. Proteins were precipitated by adding 5 volumes (1250 µL) of ice-cold acetone and incubating overnight at -20°C. Precipitates were collected by centrifugation at 13,000 rpm for 5 min at 4°C, washed twice with ice-cold methanol, and the methanol was carefully removed. Residual solvent was air-dried at room temperature for 10 min. Protein pellets were then resuspended in 65 µL of 1× SDS loading buffer and denatured by heating at 80°C for 8 min. The supernatant was collected for SDS-PAGE for and in-gel fluorescent was detected as described.

**OGT Labeling in Living Cell[4]**

HEK293T cells overexpressing HA-tagged OGT were treated with varying concentrations

of **7c** for 4 h and harvested. Cell pellets were resuspended in PBS lysis buffer (1 ‰ Triton x (polyethylene glycol mono(4-tert-octylphenyl) ether) and protease inhibitor cocktail (MedChemExpress, HY-K0010)). Lysates were subjected to three freeze-thaw cycles using liquid nitrogen, followed by centrifugation at 13,500 rpm for 15 min at 4°C. The supernatant was collected, and protein concentration was determined by BCA assay and adjusted to 2 mg/mL. 200 μL normalized samples were subsequently incubated with anti-HA magnetic beads (AlpaLifeBio, KTSM1335) (10 μL slurry per sample) in the cold room for overnight followed by washing with 0.5% PBST (PBS with 0.1% Tween-20) for 4 times. PBS (1% SDS, 30 μL) was added to each sample followed by 'click' mixture as describes above (replace the TAMRA-Azide with TAMRA-Alkynyl). The reaction was quenched by 10 μL 4× SDS loading buffer and heated at 95 °C for 5 min. The supernatant was collected for SDS-PAGE (for in-gel fluorescent) and western blot analysis.

**Immunoprecipitation**

For each IP reaction, 2.2 × $10^6$ cells HEK293T cells were seed in 10 cm dish. After cells attached, transfection reagents and plasmids were prepared and added in DMEM. After 24 h transfection, media were replaced by fresh warm complete media with DMSO or probes. After certain time of OGTACs treatment, cells were collected by cold PBS and lysed by 200 μL complete IP lysis buffer. The lysate was incubated on ice for 20 min, spun down at 14,000 rpm at 4°C for 15 mins, and the supernatant were collected for BCA assay and normalized to a final 2 mg/mL. For input, 20 μL of diluted sample was mixed with equal volume of 2× SDS-loading buffer and boiled at 95°C for 15 min. The remaining samples were subjected to HaloTrap Magnetic Agarose (Proteintech, #otma), which prebinding with protein target protein antibody. After gentle rotation at 4°C for at least 16 h, the beads were washed with IP lysis buffer and boiled in 40 μL 2X SDS-loading buffer for 5 min to elute proteins from beads. Eluted samples were directly subjected into WB analysis. To get stronger signal of RL2, we used anti-mouse-HRP (Cell signaling, #7076) as secondary antibody for RL2 and anti-rabbit-DyLight 488 (Invitrogen, #35552) or IRDye® 680RD Goat anti-Rabbit (LI-COR, 926-68071) as secondary antibody for total target proteins.

**Western blot and antibodies**

In general, cells (from 12 well plate) were lysed by 80 µL RIPA lysis buffer (Thermo Scientic 89901) supplemented with 100× protease inhibitor (MedChemExpress, HY-K0010), incubated on ice for 20 min, spun down at 14,000 rpm at 4°C for 15 min, and the supernatant were collected for BCA assay and normalizedeto a final 2 mg/mL concentration, and then the samples were denatured adding final 1× SDS-loading buffer and heating at 95°C for 5 min. About 30 µg of protein samples were loaded for SDS-PADE and blotted with indicated antibodies, with molecular weight indicated by 180 kDa pre-stained protein markers (Vazyme, MP102-01). Analysis and quantifications of western blot results were performed using Image Lab Software Version 6.1 (BioRad) or ImageJ for windows. Antibodies used in this study are as follow:

Primary antibodies: anti-RL2 (Abcam, ab2739; 1:1000 for WB), anti-CK2α (Cell Signaling Technology, 2656; 1:1000 for WB), anti-β-actin (ABclonal, AC026 or AC004; 1:100000 or 1:3000 for WB), anti-OGT (Cell Signaling Technology, 24083; 1:1000 for WB), anti-GAPDH (Santa Cruz Biotechnology, sc-47724; 1:3000 for WB), anti-Vinculin (Santa Cruz Biotechnology or ABclonal, sc-73614 or A2752; 1:1000 or 1:50000 for WB), anti-HaloTag (Promega, G9211; 1:1000 for WB); ant-HA (Vazyme, RA1004; 1:2000);
Secondary antibodies: anti-rabbit HRP-linked antibody (Cell Signaling Technology, 7074; 1:8000 for WB), anti-mouse HRP-linked antibody (Cell Signaling Technology, 7076; 1:8000 for WB; besides, we recommend a lower ratio of dilution, 1:20000, for a better detection of the RL2 signal of highly O-GlcNAcylated proteins from the whole cell lysate), anti-rabbit DyLight™ 488 antibody (ThermoFisher, 35522; 1:5000 for WB), anti-mouse DyLight™ 488 antibody (ThermoFisher, 35503; 1:5000 for WB).

**Mass Spectrometry (MS)**

For binding site identification of **7b** and **8b** (Figure 4, S4 and Table S1), purified OGT was normalized to 0.4 mg/mL in assay buffer (25 mM HEPES pH 7.5, 250 mM NaCl, 10%

glycerol). Aliquots (40 μL, 16 μg OGT) were incubated with 50 μM **7b** or **8b** (dissolved in DMSO to 5 mM stock) or DMSO control for 1 h at 25°C with 1000 rpm agitation. Proteins were precipitated with 4 times the volumes of cold acetone, pelleted by centrifugation (16,000 × g, 15 min, 4°C), and washed once with ice-cold methanol (−80°C). Precipitated proteins were resolubilized in 40 μL 6M urea/PBS (final concentration 0.4 mg/mL) and sonicated for 20 min at r.t. Sequential treatments were performed: 1) Reduction with 10 mM DTT (30 min, 37°C) 2) Alkylation with 30 mM iodoacetamide (IAA, 30 min, 35°C in darkness) 3) Quenching with 10 mM DTT (15 min, 37°C). Recombinant acetylated trypsin (rAc-Trypsin, EnzymeSource™) was reconstituted in ice-cold dd$H_2O$ to 0.5 mM. Samples were diluted with PBS to reduce urea concentration to 2 M (pH~8). Trypsin was added at 1:50 (enzyme:protein mass ratio) and digestion proceeded for 16 h at 37°C with 1000 rpm agitation. Reactions were quenched with 1% formic acid (FA), dried by centrifugal evaporation, and reconstituted in 100 μL 0.1% FA (pH~3). Peptides were desalted using Pierce™ C18 Tips (100 μL capacity) with sequential conditioning steps: 1) Activation: Methanol (1×), 80% acetonitrile (ACN)/0.1% FA (2×) 2) Equilibration: 5% ACN/0.1% FA (3×) Samples were loaded via 10 aspiration-dispersion cycles, washed with equilibration buffer (10×), and eluted with 80% ACN/0.1% FA (6×). Eluates were dried by centrifugal evaporation and stored at 4°C prior to LC-MS/MS. The data were searched against the UniProt human database using pFind, specified with trypsin digestion (allowed up to 3 missed cleavages) and lysine modification (+126.1045) as a static modification.

For the shotgun MS of OGT protein with **7b** (Figure S5), purified OGT protein (30 μg) was pre-incubated in 5 μM or 50 μM of **7b** for 1 h at r.t. in dark, and the solution mixture was vortexed every 15 min. To precipitate out the protein sample, six volumes of acetone were added to each tube and left overnight at -20°C. Afterwards, the samples were centrifuged at 8000 g at 4°C for 10 min to collect the protein pellet. The pellet was washed with 200 μL of ice cold 0.01 M HCl in 90% acetone and re-pelleted by centrifugation. To resuspend the pellet, 15 μL of 8M urea in PBS was added to the protein along with 20 μL of 1×ProteaseMax (PM) in 0.1 M ammonium bicarbonate and toped up to 100 μL with 0.1 M ammonium bicarbonate. 10 μL of freshly made 110 mM TCEP was added to the solution

and incubated for 30 min at 60°C. Then, 10 µL of 150 mM iodoacetamide solution was added and incubated for 30 min at 37°C with shaking. An additional 1.2 µL of 5×PM was added to the solution and vortexed to mix. 1.5 µL of sequencing grade Trypsin in 40 µL of supplied Trypsin buffer was added to each sample and incubated overnight at 37°C with shaking. The sample was acidified with 6 µL of FA, centrifuged at 132000 rpm for 30 min to collect the supernatant. C18 stage tips were used to desalt the peptides prior to LC-MS/MS run according to manufacturer protocol. Eluted peptides were loaded into Aurora C18 UHPLC column coupled to Bruker timTOF pro mass spectrometer. Peptides were identified and quantified with MS Fragger against UniProt human database, with trypsin digestion (allowed up to 3 missed cleavages) and lysine modification (+126.1045) as a static modification.

**Surface Plasmon Resonance (SPR)**

SPR were performed on a BiacoreTM T200 instrument (GE Healthcare). All proteins were immobilized on a CM5 chip via the typical EDC/NHS-mediated crosslinking reaction. Small molecule compounds were dissolved in DMSO and diluted in PBS at various concentrations. All experiments were performed according to the protocol provided by GE Healthcare. In each analysis, the middle concentration was duplicated at the end of the wash run to confirm the stability of the sensor surface. The parameters of SPR were set as follows: flow rate, 30 µL/min; contact time, 120s, disassociation time, 300 s. Affinity curve fitting was performed with the Biacore T200 software using a steady-state affinity model to calculate disassociation constant ($K_d$).

**Microscale thermophoresis (MST)**

Protein samples used in this assay were dialyzed against MST buffer containing 25 mM HEPES, 12.5 mM $MgCl_2$, pH 7.5. Purified OGT was fluorescently labeled using Monolith NT Protein Labeling Kit RED-NHS (Nano Temper Technologies). Serial dilution of **7a** solution was added labeled OGT and incubated on ice for 1h. The assays were conducted on Monolith NT.115 instrument (Nano Temper Technologies) at 25°C. Curve fitting and $K_\mathrm{d}$

determination were performed in MO.Affinity Analysis software.

**Molecular Docking**

Molecular docking studies were performed using the Glide module (SP mode) within the Schrödinger Suite (Version 2019). Compounds were docked into the crystal structure of OGT (PDB code: 4N3C). The protein structure was prepared for docking using the Protein Preparation Wizard suite. Ligand structures were prepared using the LigPrep suite, retaining the original chirality of the compounds. A receptor grid defining the docking site was subsequently generated. For standard docking, Glide SP mode was employed, sampling nitrogen inversions and ring conformations without imposing additional constraints. Docking poses were ranked using the GlideScore scoring function. Covalent docking was conducted using the Covalent Docking module. The specific residue targeted for covalent modification was defined, and the appropriate reaction type (covalent bond formation pattern) was selected within the Schrödinger interface. The module evaluates steric complementarity between the ligand and protein, along with the geometric feasibility of covalent bond formation, through a localized search and iterative optimization process to identify the lowest energy conformation. Covalent docking poses were ranked based on their specific scoring function. The top-ranked covalent complexes were selected for subsequent molecular dynamics simulations or experimental validation. Visualization and analysis of docking results were performed using PyMOL (Version 3.0.3) and Maestro (Version 13.9.135).

**Synthetic Method**

Unless otherwise stated, all reactions were carried out under an atmosphere of dry nitrogen in oven-dried glassware. Indicated reaction temperatures refer to those of the reaction bath, while room temperature is noted as 25°C. All solvents were anhydrous quality purchased from Meryer. Unless otherwise noted, chemical starting materials are purchased from Bide pharm or HaoYuan Chemexpress without further purification. Spectra were acquired on Bruker spectrometers: $^{1}H$ NMR: 400 or 700 MHz (recorded at 400/700 MHz); $^{13}C$ NMR:

176 MHz (recorded at 176 MHz). Chemical shifts are reported in parts-per million (ppm) relative to tetramethylsilane. Spectra were referenced according to the solvent residual peak ($CDCl_3$ 7.26 ppm, 77.0 ppm, DMSO-$d_6$ 2.50 ppm, 39.5 ppm for $^1$H, $^{13}$C, Acetone-$d_6$ 2.05 ppm, 29.8 and 206.0 ppm, respectively). Reactions were monitored by thin layer chromatography and the products were purified using fast column chromatography or preparative thin layer flash chromatography (ALUGRAM Xtra, 818333). Mass spectrometry was performed on Agilent LC-MS/MS system consisted of two Agilent 1290 series pumps and auto-sampler, coupled with 6430 triple quadrupole mass spectrometer equipped with and ESI source (Agilent Technologies, Inc., Santa Clara, CA, USA).

Compounds **2a** and **2b** were synthesized according to previously reported procedures,[5,6] using commercially available starting materials **1a** and **1b** (Bide Pharmatech Ltd.).

**7-chloro-2-oxo-1,2-dihydroquinoline-6-sulfonamide (3a)**

**2a** (300 mg, 1.08 mmol) was charged into a 25 mL round-bottom flask containing aqueous ammonia (28-30% w/w, 6 mL). The reaction mixture was heated under reflux for 3 h. The resulting suspension was filtered directly, and the filter cake was dried *in vacuo* to afford the title compound **3a** as a white solid (79.0 mg, 29% yield). $^1$H NMR (700 MHz, DMSO-$d_6$) δ 8.34 (s, 1H), 8.09 (d, $J$ = 9.6 Hz, 1H), 7.43 (s, 1H), 6.60 (d, $J$ = 9.6 Hz, 1H). $^{13}$C NMR (176 MHz, DMSO-$d_6$) δ 161.9, 141.5, 139.9, 134.2, 131.6, 129.4, 123.5, 117.2, 117.2. HRMS: calcd for $C_9H_7ClN_2O_3S$ $[M-H]^-$: 256.9793, Found: 256.9793.

**2-oxo-1,2-dihydroquinoline-6-sulfonamide (3b)**

Employing the identical synthetic route as described for **3a**, compound **2b** (300 mg, 1.34 mmol) was converted to the title compound **3b**. Isolation afforded 150 mg of a white solid in 54% yield. $^1$H NMR (700 MHz, DMSO-$d_6$) δ 12.10 (s, 1H), 7.96 (m, 3H), 7.37 (s, 3H), 6.61 (s, 1H). $^{13}$C NMR (176 MHz, DMSO-$d_6$) δ 162.1, 140.9, 140.3, 137.4, 127.5, 126.0, 123.4, 118.4, 115.8. HRMS: calcd for $C_9H_8N_2O_3S$ $[M+H]^+$: 225.0328, Found: 225.0329.

**N-((7-chloro-2-oxo-1,2-dihydroquinolin-6-yl)sulfonyl)hept-6-ynamide (5a)**

In a 25 mL round-bottom flask, **4a** (hept-6-ynoic acid, 59.3 mg, 0.47 mmol, 1.5 eq) was dissolved in anhydrous DMF (5 mL). To this solution were added EDCI (90.0 mg, 0.47 mmol, 1.5 eq), DMAP (19.5 mg, 0.16 mmol, 0.5 eq), and TEA (236 mg, 2.33 mmol, 7.5 eq). The mixture was stirred at room temperature for 30 min, whereupon **3a** (80.0 mg, 0.31 mmol, 1.0 eq) was added. After stirring 48 h at room temperature, the solvent was removed *in vacuo*. Purification by flash column chromatography (DCM/MeOH, 50:1 v/v) afforded the **5a** as a white solid (37.9 mg, 33% yield). $^{1}H$ NMR (700 MHz, DMSO-$d_6$)δ 12.48 (s, 1H), 12.16 (s, 1H), 8.49 (s, 1H), 8.16 (d, *J* = 9.6 Hz, 1H), 7.43 (s, 1H), 6.64 (dd, *J* = 9.6, 1.5 Hz, 1H), 2.74 (t, *J* = 2.6 Hz, 1H), 2.25 (t, *J* = 7.3 Hz, 2H), 2.12 – 2.05 (m, 2H), 1.53 – 1.42 (m, 2H), 1.34 – 1.26 (m, 2H). $^{13}C$ NMR (176 MHz, DMSO-$d_6$) δ 171.6, 161.8, 142.6, 139.8, 133.1, 131.4, 129.2, 123.7, 117.3, 117.3, 84.1, 71.4, 34.8, 27.2, 23.3, 17.4. HRMS: calcd for $C_{16}H_{15}ClN_2O_4S$ $[M+Na]^+$: 389.0333, Found: 389.0328.

**N-((2-oxo-1,2-dihydroquinolin-6-yl)sulfonyl)hept-6-ynamide (6a)**

Employing the procedure analogous to that described for **5a** but starting from **3b** (250 mg, 1.0 mmol, 1.0 eq) and **4a** (195 mg, 1.54 mmol, 1,5 eq), the title compound **6a** was obtained as a white solid (163 mg, 48% yield). $^{1}H$ NMR (700 MHz, DMSO-$d_6$) δ 12.17 (s, 1H), 12.09 (s, 1H), 8.28 (d, *J* = 1.4 Hz, 1H), 8.12 (d, *J* = 9.8 Hz, 1H), 7.94 (dd, *J* = 9.4, 2.1 Hz, 1H), 7.44 (d, *J* = 2.1 Hz, 1H), 6.62 (dd, *J* = 9.8, 1.4 Hz, 1H), 2.73 (t, *J* = 2.6 Hz, 1H), 2.20 (t, *J* = 7.4 Hz, 2H), 2.11 – 2.03 (m, 2H), 1.52 – 1.41 (m, 2H), 1.34 – 1.26 (m, 2H). $^{13}C$ NMR (176 MHz, DMSO-$d_6$) δ 174.1, 164.7, 144.7, 142.8, 134.7, 131.5, 131.3, 126.2, 121.0, 118.5, 86.8, 74.1, 37.4, 29.8, 25.8, 20.0. HRMS: calcd for $C_{16}H_{16}N_2O_4S$ $[M+Na]^+$: 355.0723, Found: 355.0721.

**N-((7-chloro-2-oxo-1,2-dihydroquinolin-6-yl)sulfonyl)octanamide (5b)**

Using **3a** (100mg, 0.39 mmol, 1.0 eq) and **4b** (octanoic acid, 86.5 mg, 0.60 mmol, 1.5 eq) as starting materials via the synthetic route analogous to that of **5a**, the compound **5b** was obtained as a white solid (97.0 mg, 65% yield) after purification. $^{1}H$ NMR (700 MHz, DMSO-

*d*6) δ 12.44 (s, 1H), 12.17 (s, 1H), 8.49 (s, 1H), 8.16 (d, *J* = 9.6 Hz, 1H), 7.42 (s, 1H), 6.63 (dd, *J* = 9.7, 1.1 Hz, 1H), 2.22 (t, *J* = 7.2 Hz, 2H), 1.41 – 1.36 (m, 2H), 1.20 – 1.11 (m, 6H), 1.10 – 1.05 (m, 2H), 0.80 (t, *J* = 7.1 Hz, 3H). $^{13}$C NMR (176 MHz, DMSO-$d_6$) δ 171.7, 161.8, 142.6, 139.8, 133.2, 131.5, 129.1, 123.7, 117.3, 117.2, 35.3, 31.1, 28.3, 28.2, 24.1, 22.0, 14.0. HRMS: calcd for $C_{17}H_{21}ClN_2O_4S$ [M-H]$^-$: 383.0837, Found: 383.0837.

**N-((2-oxo-1,2-dihydroquinolin-6-yl)sulfonyl)octanamide (6b)**

Using **3b** (152 mg, 0.62 mmol, 1.0 eq) and **4b** (octanoic acid, 135 mg, 0.93 mmol, 1.5 eq) as starting materials via the synthetic route analogous to that of **5a**, the compound **6b** was obtained as a white solid (143 mg, 68% yield) after purification. $^1$H NMR (700 MHz, DMSO-$d_6$) δ 12.17 (s, 1H), 12.05 (s, 1H), 8.28 (d, *J* = 1.7 Hz, 1H), 8.13 (d, *J* = 9.6 Hz, 1H), 7.94 (dd, *J* = 8.7, 1.9 Hz, 1H), 7.43 (d, *J* = 8.7 Hz, 1H), 6.62 (dd, *J* = 9.6, 1.7 Hz, 1H), 2.17 (t, *J* = 7.2 Hz, 2H), 1.41 – 1.31 (m, 2H), 1.21 – 1.01 (m, 8H), 0.79 (t, *J* = 7.2 Hz, 3H). $^{13}$C NMR (176 MHz, DMSO-$d_6$) δ 174.4, 164.7, 144.7, 142.8, 134.8, 131.5, 131.3, 126.1, 121.0, 118.4, 37.9, 33.7, 30.9, 30.8, 26.7, 24.6, 16.6. HRMS: calcd for $C_{17}H_{22}N_2O_4S$ [M-H]$^-$: 349.1227, Found: 349.1227.

**6-azido-N-((7-chloro-2-oxo-1,2-dihydroquinolin-6-yl)sulfonyl)hexanamide (5c)**

Using **3a** (50.0 mg, 0.19 mmol, 1.0 eq) and **4c** (6-azidohexanoic acid, 45.0 mg, 0.29 mmol, 1.5 eq) as starting materials via the synthetic route analogous to that of **5a**, the **5c** was obtained as an off-white solid (44.1 mg, 57% yield) after purification. $^1$H NMR (700 MHz, DMSO-$d_6$) δ 12.46 (s, 1H), 12.16 (s, 1H), 8.49 (s, 1H), 8.15 (d, *J* = 9.7 Hz, 1H), 7.43 (s, 1H), 6.63 (d, *J* = 9.6 Hz, 1H), 3.24 (t, *J* = 6.9 Hz, 2H), 2.25 (t, *J* = 7.3 Hz, 2H), 1.46 – 1.38 (m, 4H), 1.23 – 1.12 (m, 2H). $^{13}$C NMR (176 MHz, DMSO-$d_6$) δ 171.6, 161.8, 142.6, 139.8, 133.2, 131.5, 129.1, 123.7, 117.3, 117.3, 50.5, 35.2, 27.9, 25.5, 23.6. HRMS: calcd for $C_{15}H_{16}ClN_5O_4S$ [M+Na]$^+$: 420.0503, Found: 420.0504.

**N-((7-chloro-2-oxo-1,2-dihydroquinolin-6-yl)sulfonyl)-N-(cyanomethyl)hept-6-ynamide (7a)**

A solution of **5a** (43.0 mg, 0.12 mmol, 1.0 eq) and iodoacetonitrile (30.0 mg, 0.18 mmol, 1.5 eq) in anhydrous DMF (3 mL) was prepared in a 10 mL round-bottom flask. DIPEA (60.7 mg, 0.47 mmol, 4.0 eq) was added, and the reaction mixture was stirred at room temperature for 48 h. The solvent was removed *in vacuo*. Purification by flash column chromatography (hexane/EtOAc, 1:1 to 1:3 v/v gradient) afforded the title compound **7a** as a white solid (13.0 mg, 27% yield). $^1$H NMR (400 MHz, DMSO-$d_6$) δ 12.26 (s, 1H), 8.65 (s, 1H), 8.16 (d, *J* = 9.7 Hz, 1H), 7.47 (d, *J* = 8.1 Hz, 1H), 6.68 (d, *J* = 9.6 Hz, 1H), 4.95 (s, 2H), 2.71 (dd, *J* = 8.4, 5.2 Hz, 2H), 2.08 (td, *J* = 6.9, 2.5 Hz, 2H), 1.59 – 1.48 (m, 2H), 1.40 – 1.31 (m, 2H). $^{13}$C NMR (176 MHz, DMSO-$d_6$) δ 172.1, 161.8, 143.4, 139.7, 133.6, 131.4, 127.4, 124.0, 117.8, 117.6, 116.1, 84.0, 71.5, 34.8, 33.9, 27.0, 23.1, 17.4. HRMS: calcd for $C_{18}H_{16}ClN_3O_4S$ [M+Na]$^+$: 428.0442, Found: 428.0441.

**N-(cyanomethyl)-N-((2-oxo-1,2-dihydroquinolin-6-yl)sulfonyl)hept-6-ynamide (8a)**

Following the procedure analogous to that of **7a** but starting from **6a** (50.0 mg, 0.15 mmol, 0.15 eq), the title compound **8a** was obtained as a white solid (30.0 mg, 54% yield) after purification. $^1$H NMR (400 MHz, DMSO-$d_6$) δ 12.24 (s, 1H), 8.47 (d, *J* = 1.9 Hz, 1H), 8.11 – 8.03 (m, 2H), 7.48 (d, *J* = 8.8 Hz, 1H), 6.67 (d, *J* = 9.6 Hz, 1H), 4.92 (s, 2H), 2.71 (d, *J* = 6.5 Hz, 3H), 2.09 (m, 2H), 1.58 – 1.49 (m, 2H), 1.42 – 1.33 (m, 2H). $^{13}$C NMR (176 MHz, DMSO-$d_6$) δ 172.0, 162.0, 142.8, 140.0, 130.3, 129.3, 128.8, 123.9, 118.7, 116.5, 116.4, 84.2, 71.5, 34.7, 34.0, 27.0, 23.1, 17.4. HRMS: calcd for $C_{18}H_{17}N_3O_4S$ [M+Na]$^+$: 394.0832, Found: 394.0829.

**N-((7-chloro-2-oxo-1,2-dihydroquinolin-6-yl)sulfonyl)-N-(cyanomethyl)octanamide (7b)**

Following the procedure analogous to that of **7a** but starting from **5b** (60.0 mg, 0.16 mmol, 1.0 eq), the title compound **7b** was obtained as a white solid (17.0 mg, 26% yield) after purification. $^1$H NMR (700 MHz, DMSO-$d_6$) δ 12.29 (s, 1H), 8.66 (s, 1H), 8.17 (d, *J* = 9.7 Hz, 1H), 7.48 (s, 1H), 6.69 (d, *J* = 9.7 Hz, 1H), 4.96 (s, 2H), 2.66 (t, *J* = 7.2 Hz, 2H), 1.43 – 1.37 (m, 2H), 1.19 – 1.05 (m, 8H), 0.80 (t, *J* = 7.2 Hz, 3H). $^{13}$C NMR (176 MHz, DMSO-

*d*6) δ 172.2, 161.8, 143.4, 139.7, 133.6, 131.5, 127.5, 124.1, 117.8, 117.7, 116.2, 35.2, 33.9, 31.1, 28.2, 28.0, 24.0, 22.0, 14.0. HRMS: calcd for $C_{19}H_{22}ClN_3O_4S$ $[M+Na]^+$: 446.0911, Found: 446.0909.

**N-(cyanomethyl)-N-((2-oxo-1,2-dihydroquinolin-6-yl)sulfonyl)octanamide (8b)**

Following the procedure analogous to that of **7a** but starting from **6b** (50.0 mg, 0.19 mmol, 1.0 eq), the title compound **8b** was obtained as a white solid (18.0 mg, 55% yield) after purification. $^1H$ NMR (700 MHz, DMSO-$d_6$) δ 12.26 (s, 1H), 8.47 (d, *J* = 1.8 Hz, 1H), 8.11 – 8.04 (m, 2H), 7.48 (d, *J* = 8.8 Hz, 1H), 6.67 (d, *J* = 9.6 Hz, 1H), 4.94 (s, 2H), 2.65 (t, *J* = 7.1 Hz, 2H), 1.44 – 1.38 (m, 2H), 1.20 – 1.00 (m, 8H), 0.79 (t, *J* = 7.2 Hz, 3H). $^{13}C$ NMR (176 MHz, DMSO-$d_6$) δ 172.1, 162.0, 142.8, 140.0, 130.4, 129.2, 128.7, 124.0, 118.7, 116.5, 116.4, 35.0, 34.0, 31.1, 28.3, 28.0, 24.0, 22.0, 14.0. HRMS: calcd for $C_{19}H_{23}N_3O_4S$ $[M+Na]^+$: 412.1301, Found: 412.1300.

**6-azido-N-((7-chloro-2-oxo-1,2-dihydroquinolin-6-yl)sulfonyl)-N-(cyanomethyl)hexanamide (7c)**

Following the procedure analogous to that of **7a** but starting from **5c** (20.0 mg, 0.05 mmol, 1.0 eq), the title compound **7c** was obtained as a white solid (7.8 mg, 35% yield) after purification. $^1H$ NMR (700 MHz, Acetone-$d_6$) δ 11.23 (s, 1H), 8.64 (s, 1H), 8.21 – 8.06 (m, 1H), 7.64 (s, 1H), 6.77 – 6.64 (m, 1H), 3.37 – 3.20 (m, 2H), 2.89 (d, *J* = 10.6 Hz, 2H), 2.80 (dd, *J* = 9.3, 5.3 Hz, 2H), 1.61 – 1.55 (m, 2H), 1.52 – 1.46 (m, 2H), 1.30 (ddd, *J* = 15.4, 8.3, 4.9 Hz, 2H). $^{13}C$ NMR (176 MHz, Acetone-$d_6$) δ 172.8, 162.3, 144.6, 140.4, 134.4, 133.3, 129.2, 125.1, 118.9, 118.8, 116.3, 51.7, 36.5, 34.6, 29.1, 26.5, 24.6. HRMS: calcd for $C_{17}H_{17}N_6O_4S$ $[M+Na]^+$: 459.0612, Found: 459.0610.

**N-(phenylsulfonyl)hept-6-ynamide (FR01)**

To a solution of benzenesulfonamide (100 mg, 0.63 mmol, 1.0 eq) in anhydrous DMF (2 mL) was added 6-heptynoic acid (96 mg, 0.76 mmol, 1.2 eq), EDCI (180 mg, 0.94 mmol, 1.5 eq), DMAP (38 mg, 0.31 mmol, 0.5 eq), and triethylamine (476 mg, 4.7 mmol, 7.5 eq)

sequentially. The reaction mixture was stirred at r.t. for overnight. Upon completion, the solvent was removed under reduced pressure. The crude residue was purified by flash column chromatography on silica gel (eluent: hexane/ethyl acetate = 3/2) to afford the title compound **FR01** as a white solid (82 mg, 49.3% yield). $^1$H NMR (400 MHz, $CDCl_3$) δ 8.07 (dd, *J* = 8.3, 0.9 Hz, 2H), 7.66 (m, 1H), 7.56 (dd, *J* = 10.6, 4.8 Hz, 2H), 2.30 (t, *J* = 7.4 Hz, 2H), 2.14 (m, 2H), 1.92 (t, *J* = 2.6 Hz, 1H), 1.75 – 1.61 (m, 2H), 1.51 – 1.37 (m, 2H). $^{13}$C NMR (176 MHz, $CDCl_3$) δ 170.8, 138.6, 134.2, 129.2 (2), 128.4 (2), 83.8, 69.0, 35.8, 27.5, 23.4, 18.2. HRMS: calcd for $C_{13}H_{15}NO_3S$ $[M+Na]^+$: 288.0665, Found: 288.0661.

**N-(cyanomethyl)-N-(phenylsulfonyl)hept-6-ynamide (FR02)**

A mixture of **FR01** (40 mg, 0.15 mmol, 1.0 eq) and iodoacetonitrile (102 mg, 0.6 mmol, 4.0 eq) in anhydrous DMF (1 mL) was treated with DIPEA (155 mg, 1.2 mmol, 8.0 eq). The reaction was stirred at r.t. for 48 h. After the solvent was removed under reduced pressure, the crude product was purified by flash column chromatography on silica gel (eluent: hexane/ethyl acetate = 3/1) to afford the title compound **FR02** as a yellow oil (20 mg, 43.8% yield). $^1$H NMR (400 MHz, $CDCl_3$) δ 8.02 – 7.95 (m, 2H), 7.78 – 7.71 (m, 1H), 7.69 – 7.61 (m, 2H), 4.77 (s, 2H), 2.70 (t, *J* = 7.3 Hz, 1H), 2.45 (t, *J* = 7.4 Hz, 1H), 2.27 – 2.19 (m, 1H), 2.18 – 2.09 (m, 1H), 2.01 – 1.89 (m, 1H), 1.74 (m, 2H), 1.62 – 1.54 (m, 1H), 1.51 – 1.41 (m, 1H). $^{13}$C NMR (176 MHz, $CDCl_3$) δ 171.8, 138.4, 135.0, 127.7 (2), 114.8, 83.8, 69.0, 48.3, 35.6, 33.1, 27.5, 23.5, 18.2. HRMS: calcd for $C_{15}H_{16}N_2O_3S$ $[M+Na]^+$: 327.0774, Found: 327.0770.

**Synthesis of probe S1**:

To a solution of 2-[2-[(6-chlorohexyl)oxy]ethoxy]acetic acid (18.0 mg, 0.075 mmol, 1.2 eq) in anhydrous DMF (1 mL) contained in a 10 mL round-bottom flask, HATU (35.9 mg, 0.095 mmol, 1.5 eq) and DIPEA (24.4 mg, 0.19 mmol, 3.0 eq) were added sequentially at room temperature. The resulting mixture was stirred at room temperature for 30 min. DBCO-PEG3-amine (30.2 mg, 0.063 mmol, 1.0 eq) was then added, and stirring was continued

at room temperature overnight. The reaction mixture was diluted with water and extracted with ethyl acetate (3 × 15 mL). The combined organic extracts were washed with saturated brine (1 × 15 mL), dried over anhydrous $Na_2SO_4$, and concentrated under reduced pressure. The crude residue was adsorbed onto silica gel and purified by flash column chromatography using a gradient elution of dichloromethane/methanol (50:1 to 25:1, v/v) to afford the product **S1** as a pale yellow oily solid (35.0 mg, 79%). $^1$H NMR (700 MHz, Acetone-$d_6$) δ 7.68 (m, 2H), 7.51 – 7.43 (m, 4H), 7.40 – 7.33 (m, 2H), 7.28 (m, 1H), 7.14 (s, 1H), 5.12 (d, *J* = 14.0 Hz, 1H), 3.94 – 3.91 (m, 2H), 3.67 – 3.62 (m, 3H), 3.61 – 3.56 (m, 10H), 3.54 – 3.50 (m, 4H), 3.46 (m, 2H), 3.43 – 3.40 (m, 3H), 3.26 (m, 2H), 2.77 – 2.71 (m, 1H), 2.37 (m, 1H), 2.16 – 2.11 (m, 1H), 1.92 – 1.86 (m, 1H), 1.79 – 1.74 (m, 2H), 1.61 – 1.56 (m, 2H), 1.48 – 1.42 (m, 3H), 1.38 (m, 2H).$^{13}$C NMR (176 MHz, Acetone-$d_6$) δ 172.7, 172.3, 170.8, 152.9, 149.5, 133.4, 130.5, 129.6, 128.9, 128.8, 128.4, 127.7, 126.0, 124.0, 123.0, 115.1, 108.9, 71.6, 71.6, 71.0, 70.9, 70.7, 70.7, 70.7, 70.6, 55.9, 45.8, 39.8, 39.6, 39.2, 39.1, 33.3, 31.5, 30.7, 30.3, 27.3, 26.1. HRMS: calcd for $C_{37}H_{50}ClN_3O_8$ $[M+Na]^+$: 722.3178, Found: 722.3172.

(1) Ma, B.; Khan, K. S.; Xu, T.; Xeque Amada, J.; Guo, Z.; Huang, Y.; Yan, Y.; Lam, H.; Cheng, A. S. L.; Ng, B. W. L. Targeted Protein O-GlcNAcylation Using Bifunctional Small Molecules. *J Am Chem Soc* **2024**, *146* (14), 9779–9789. https://doi.org/10.1021/jacs.3c14380.

(2) Chong, P. A.; Nosella, M. L.; Vanama, M.; Ruiz-Arduengo, R.; Forman-Kay, J. D. Exploration of O-GlcNAc Transferase Glycosylation Sites Reveals a Target Sequence Compositional Bias. *Journal of Biological Chemistry* **2023**, *299* (5). https://doi.org/10.1016/j.jbc.2023.104629.

(3) Hirata, T.; Nagae, M.; Osuka, R. F.; Mishra, S. K.; Yamada, M.; Kizuka, Y. Recognition of Glycan and Protein Substrates by N-Acetylglucosaminyltransferase-V. *Biochim Biophys Acta Gen Subj* **2020**, *1864* (12). https://doi.org/10.1016/j.bbagen.2020.129726.

(4) Liu, Z.; Remsberg, J. R.; Li, H.; Njomen, E.; Demeester, K. E.; Tao, Y.; Xia, G.; Hayward, R. E.; Yoo, M.; Nguyen, T.; Simon, G. M.; Schreiber, S. L.; Melillo, B.; Cravatt, B. F. *Proteomic Ligandability Maps of Spirocycle Acrylamide Stereoprobes Identify Covalent ERCC3 Degraders*. *J Am Chem Soc* **2024**, *146* (15), 10393-10406. https://pubs.acs.org/doi/10.1021/jacs.3c13448.

(5) Martin, S. E. S.; Tan, Z. W.; Itkonen, H. M.; Duveau, D. Y.; Paulo, J. A.; Janetzko, J.; Boutz, P. L.; Törk, L.; Moss, F. A.; Thomas, C. J.; Gygi, S. P.; Lazarus, M. B.; Walker, S. Structure-Based Evolution of Low Nanomolar O-GlcNAc Transferase Inhibitors. *J Am Chem Soc* **2018**, *140* (42), 13542–13545. https://doi.org/10.1021/jacs.8b07328.

(6) Ortiz-Meoz, R. F.; Jiang, J.; Lazarus, M. B.; Orman, M.; Janetzko, J.; Fan, C.; Duveau, D. Y.; Tan, Z. W.; Thomas, C. J.; Walker, S. A Small Molecule That Inhibits OGT Activity in Cells. *ACS Chem Biol* **2015**, *10* (6), 1392–1397. https://doi.org/10.1021/acschembio.5b00004.

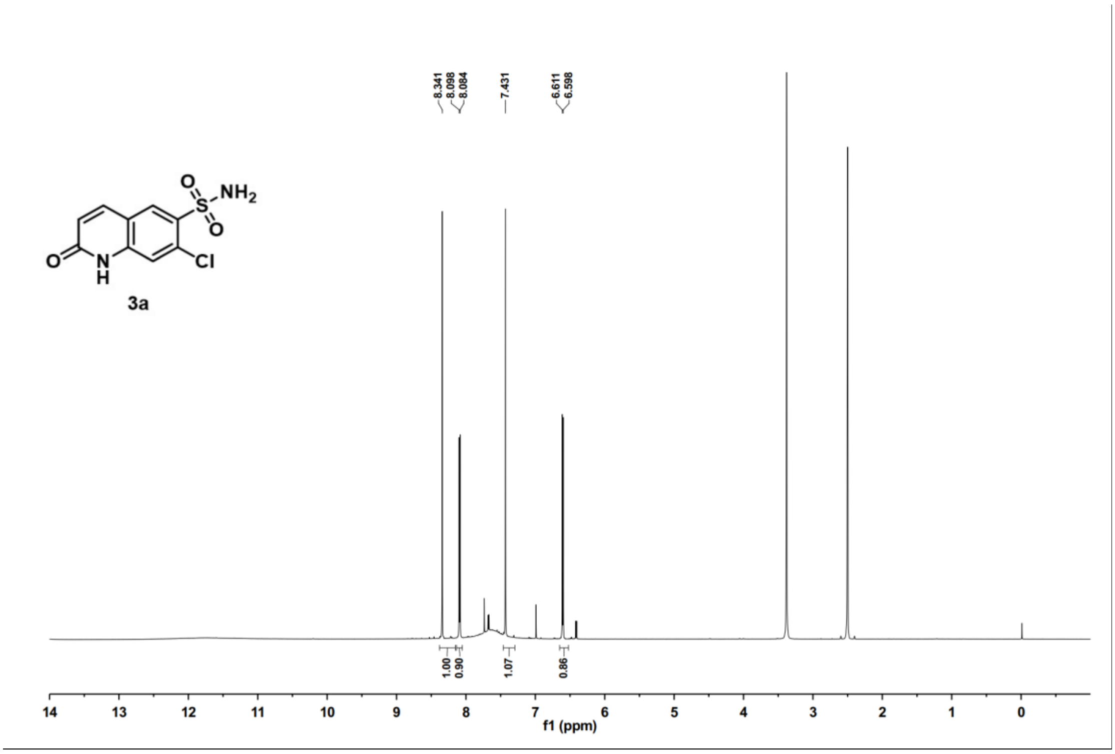
8.341
8.098
8.084
7.431
6.611
6.598
NH2
Cl
O
N
H
1.00
0.90
1.07
0.86
f1 (ppm)

3a

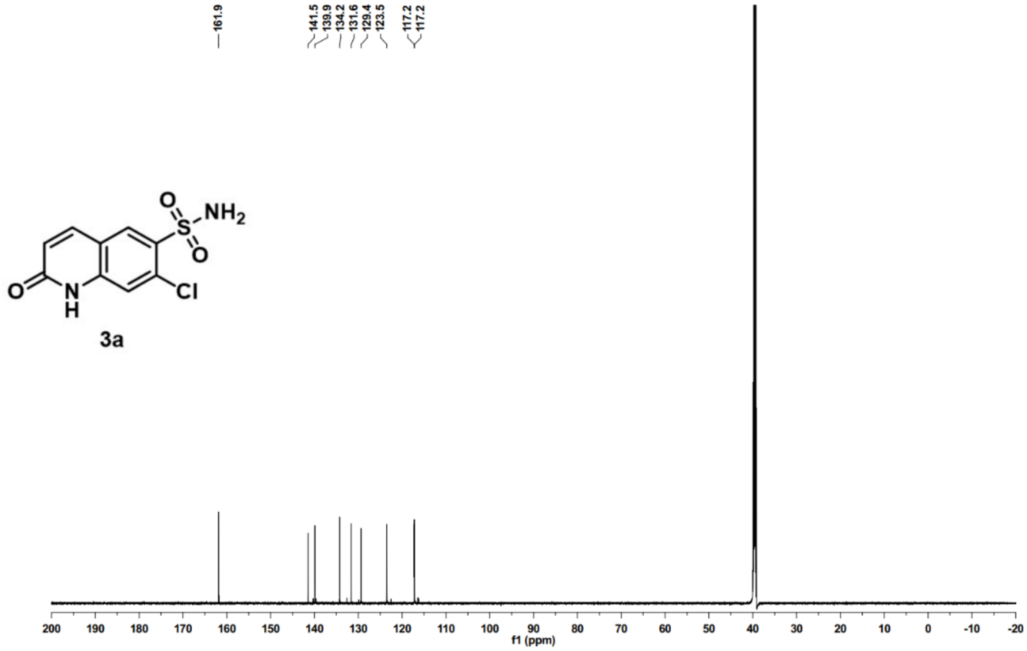
161.9
141.5
139.9
134.2
131.6
129.4
123.5
117.2
117.2
NH2
Cl
O
N
H
f1 (ppm)

3a

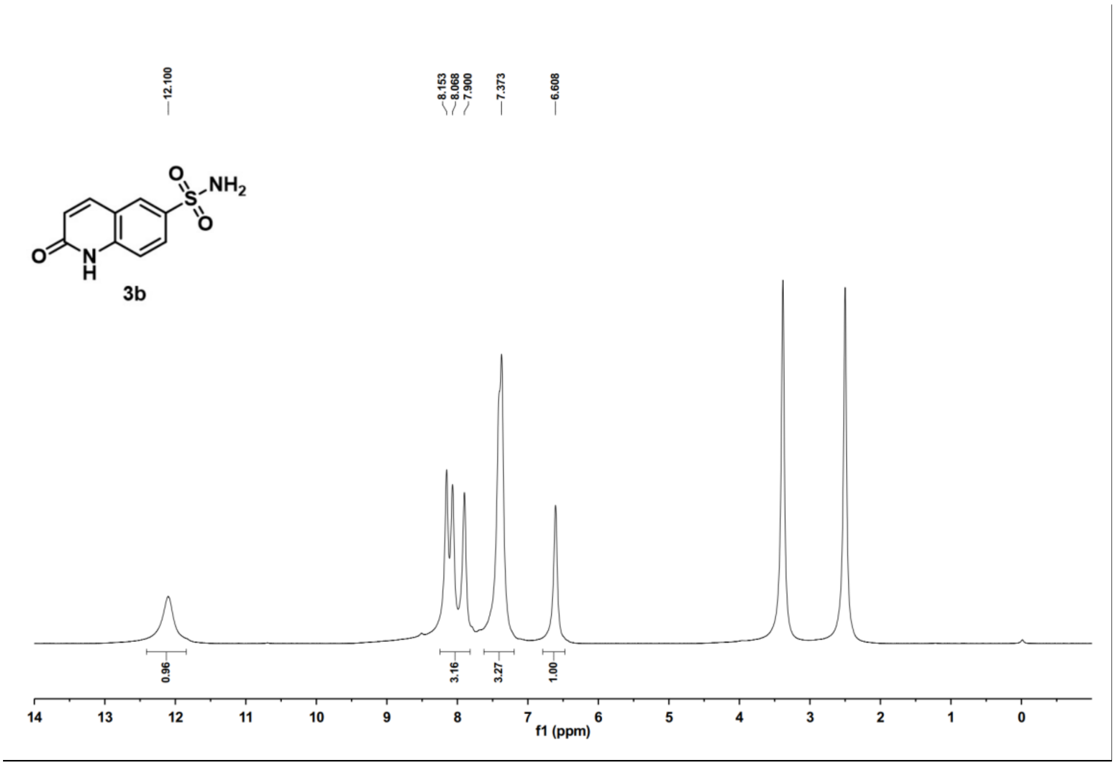
12.100
8.153
8.068
7.900
7.373
6.608
NH2
3b
0.96
3.16
3.27
1.00
14 13 12 11 10 9 8 7 6 5 4 3 2 1 0
f1 (ppm)

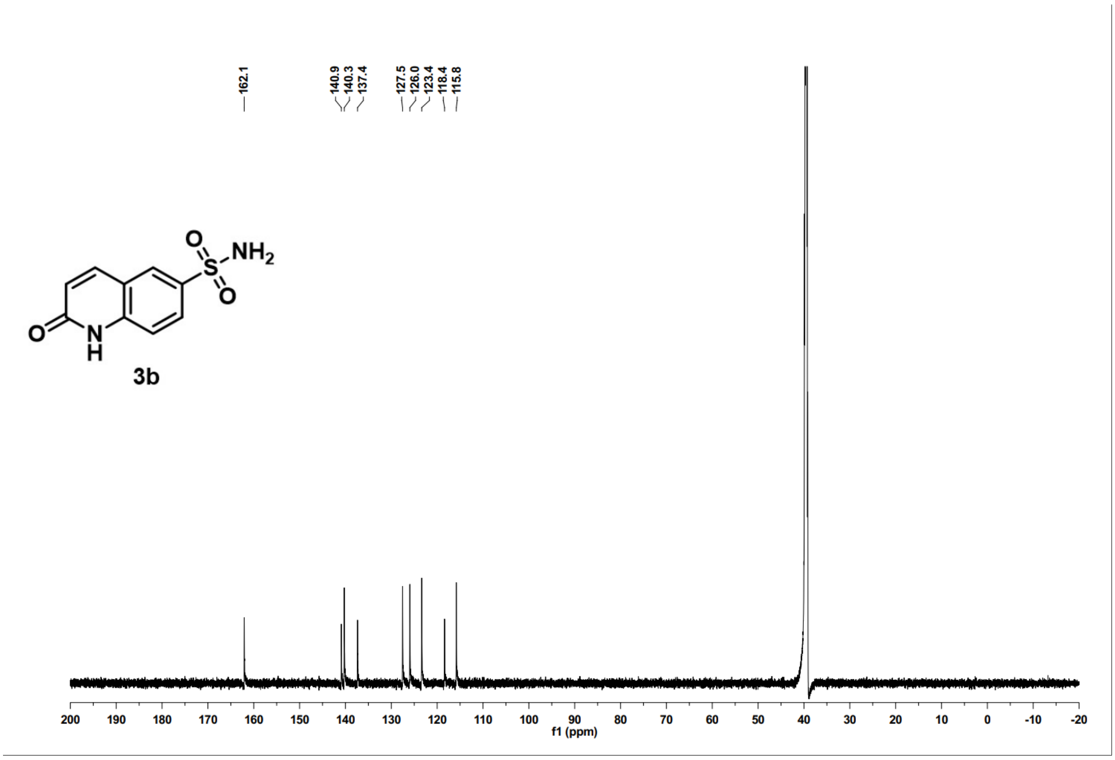
162.1
140.9
140.3
137.4
127.5
126.0
123.4
118.4
115.8
NH2
3b
200 190 180 170 160 150 140 130 120 110 100 90 80 70 60 50 40 30 20 10 0 -10 -20
f1 (ppm)

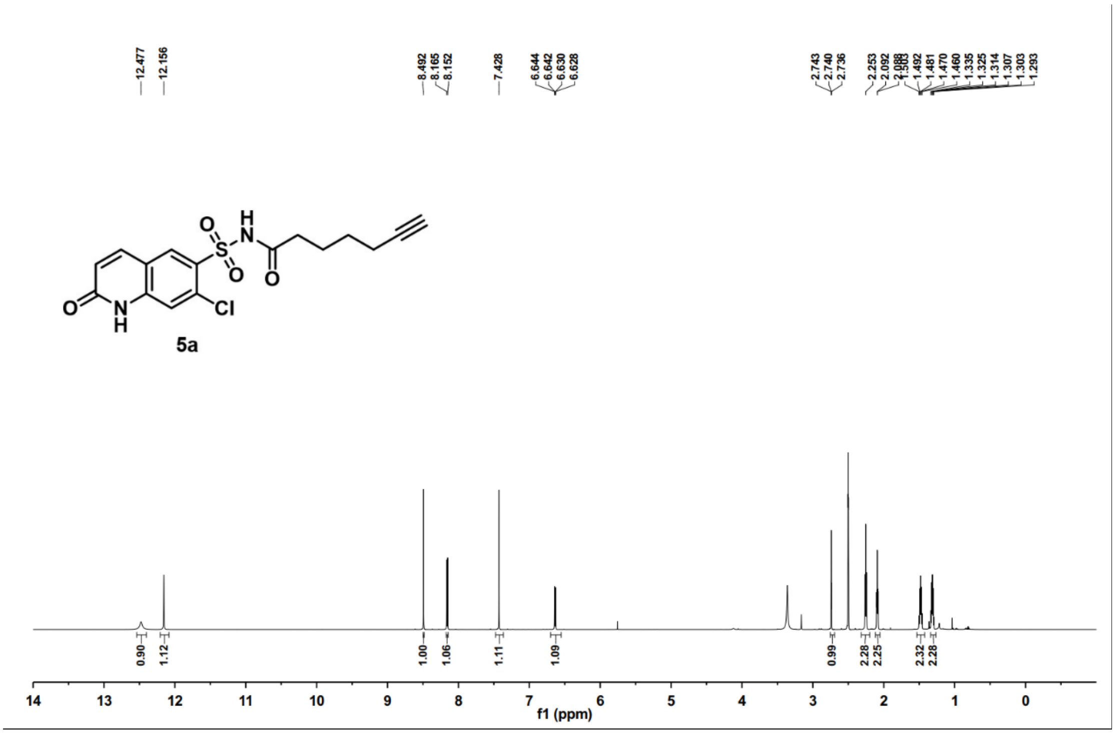
12.477
12.156
8.492
8.165
8.152
7.428
6.644
6.642
6.630
6.628
2.743
2.740
2.736
2.253
2.092
2.088
1.503
1.492
1.481
1.470
1.460
1.335
1.325
1.314
1.307
1.303
1.293
5a
0.90
1.12
1.00
1.06
1.11
1.09
0.99
2.28
2.25
2.32
2.28
f1 (ppm)

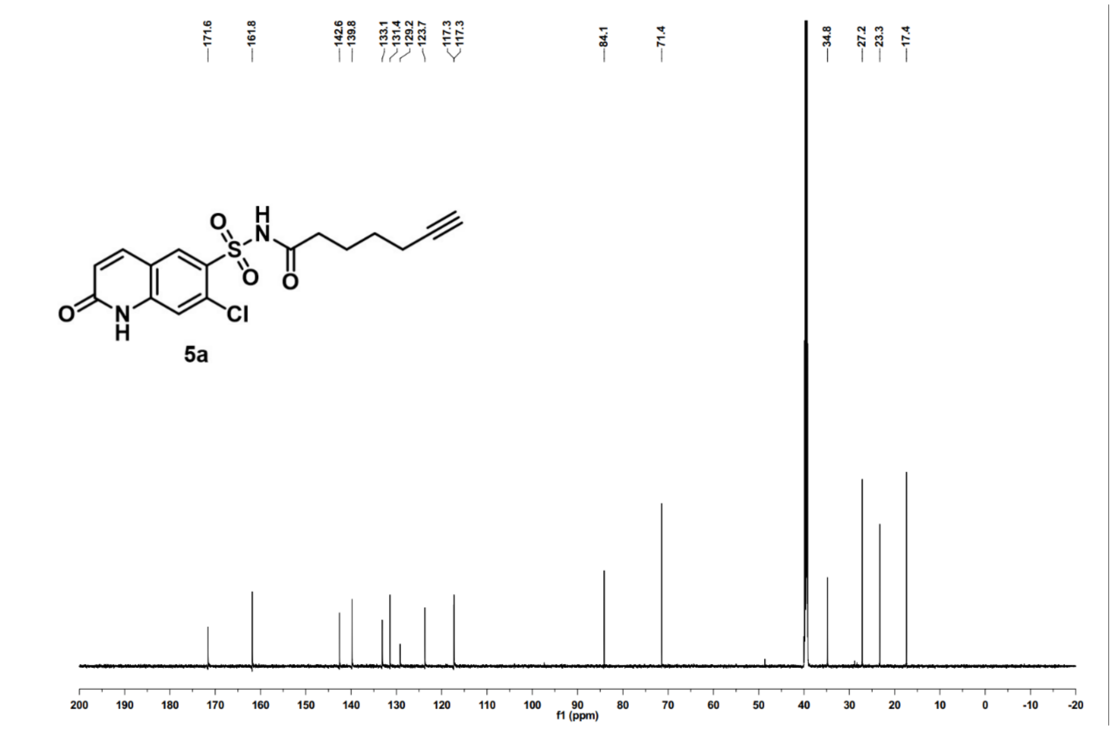
171.6
161.8
142.6
139.8
133.1
131.4
129.2
123.7
117.3
117.3
84.1
71.4
34.8
27.2
23.3
17.4
5a
f1 (ppm)

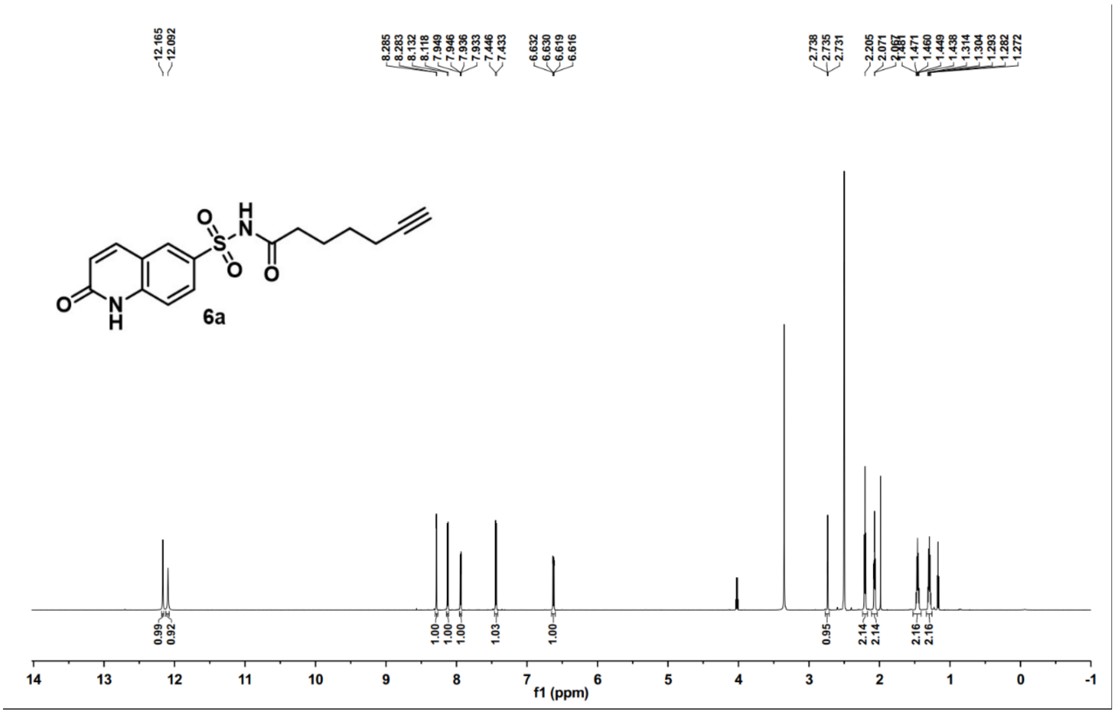
12.165
12.092
8.285
8.283
8.132
8.118
7.949
7.946
7.936
7.933
7.446
7.433
6.632
6.630
6.619
6.616
2.738
2.735
2.731
2.205
2.071
1.471
1.460
1.449
1.438
1.314
1.304
1.293
1.282
1.272
0.99
0.92
1.00
1.00
1.00
1.03
1.00
0.95
2.14
2.14
2.16
2.16
6a
f1 (ppm)

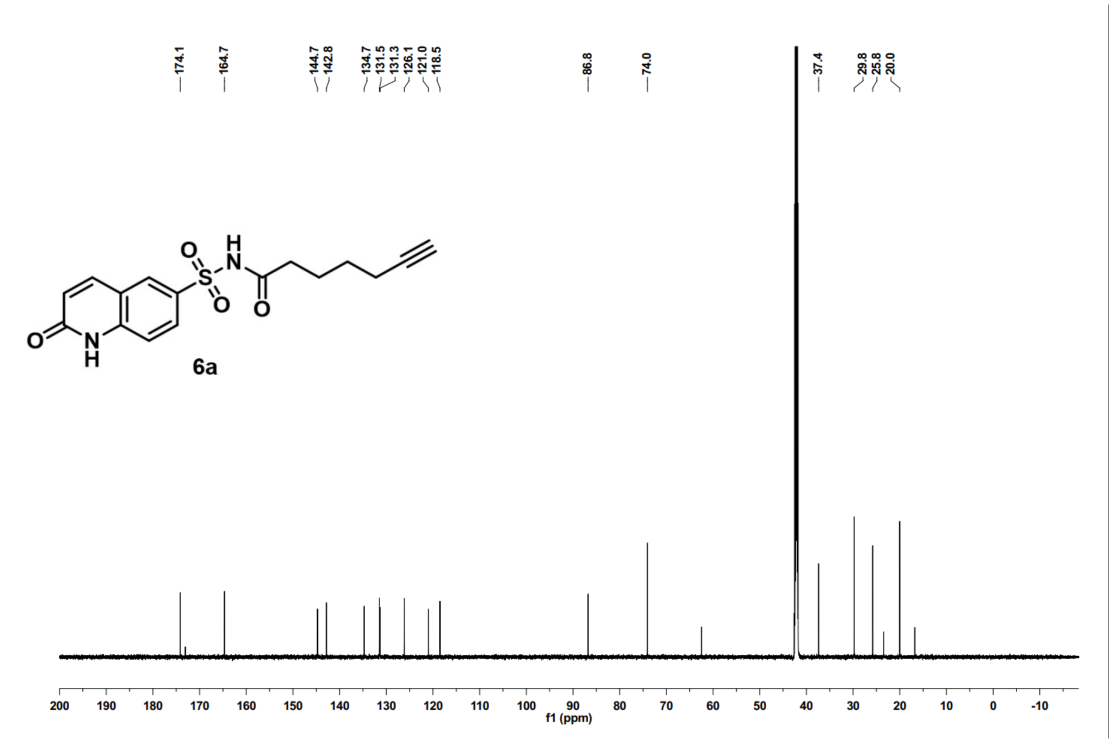
174.1
164.7
144.7
142.8
134.7
131.5
131.3
126.1
121.0
118.5
86.8
74.0
37.4
29.8
25.8
20.0
6a
f1 (ppm)

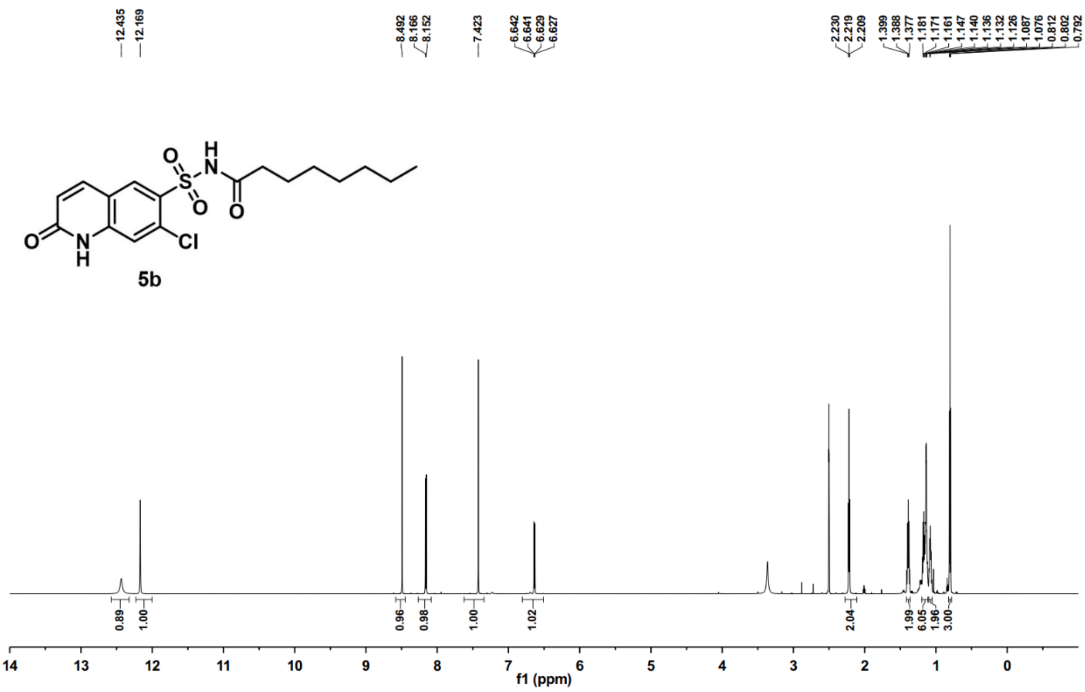
5b
12.435
12.169
8.492
8.166
8.152
7.423
6.642
6.641
6.629
6.627
2.230
2.219
2.209
1.399
1.388
1.377
1.181
1.171
1.161
1.147
1.140
1.136
1.132
1.126
1.087
1.076
0.812
0.802
0.792
0.89
1.00
0.96
0.98
1.00
1.02
2.04
1.99
6.05
1.96
3.00
f1 (ppm)

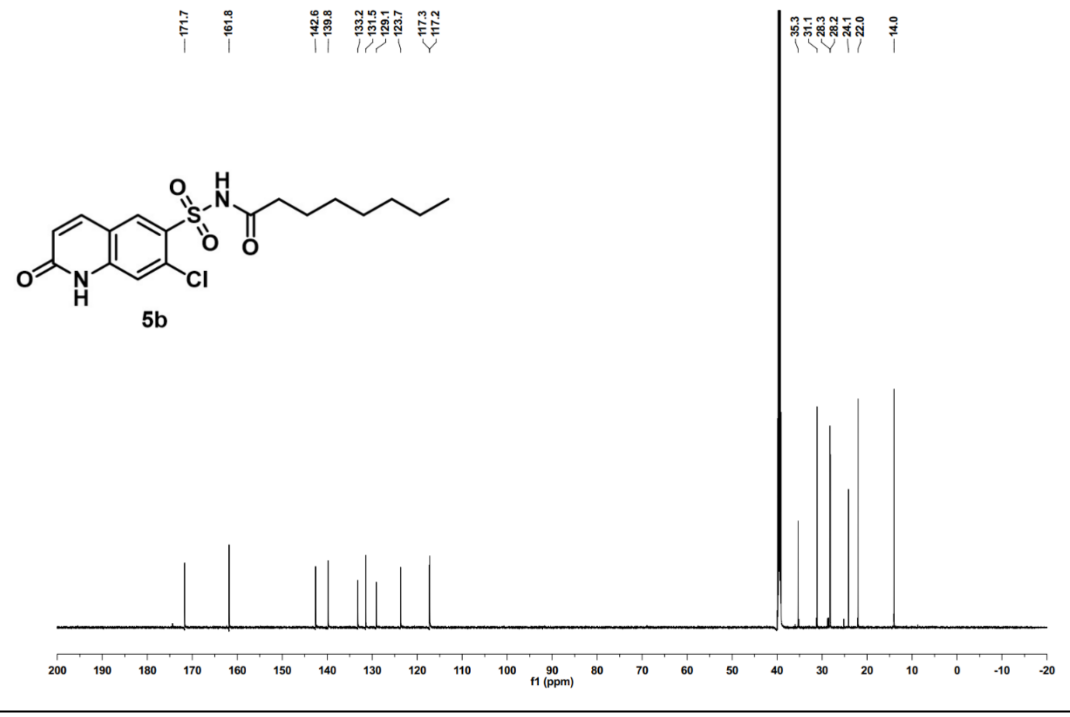
5b
171.7
161.8
142.6
139.8
133.2
131.5
129.1
123.7
117.3
117.2
35.3
31.1
28.3
28.2
24.1
22.0
14.0
f1 (ppm)

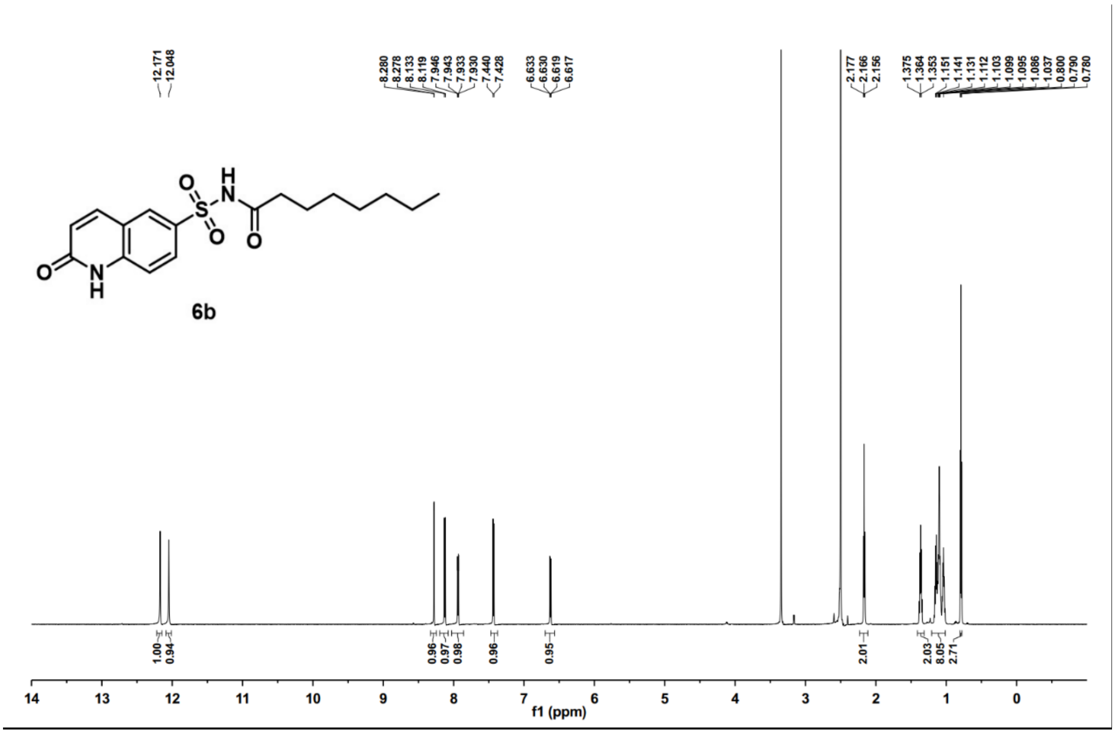

12.171
12.048
8.280
8.278
8.133
8.119
7.946
7.943
7.933
7.930
7.440
7.428
6.633
6.630
6.619
6.617
2.177
2.166
2.156
1.375
1.364
1.353
1.151
1.141
1.131
1.112
1.103
1.099
1.095
1.086
1.037
0.800
0.790
0.780
6b
1.00
0.94
0.96
0.97
0.98
0.96
0.95
2.01
2.03
8.05
2.71
f1 (ppm)


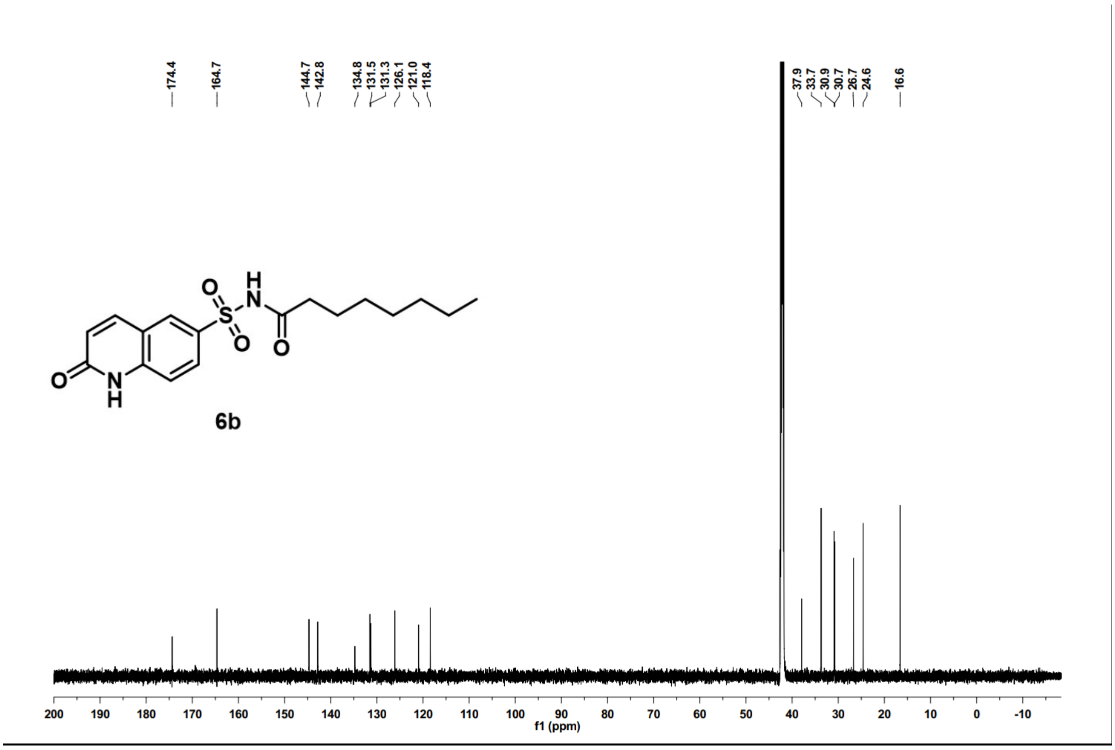

174.4
164.7
144.7
142.8
134.8
131.5
131.3
126.1
121.0
118.4
37.9
33.7
30.9
30.7
26.7
24.6
16.6
6b
f1 (ppm)

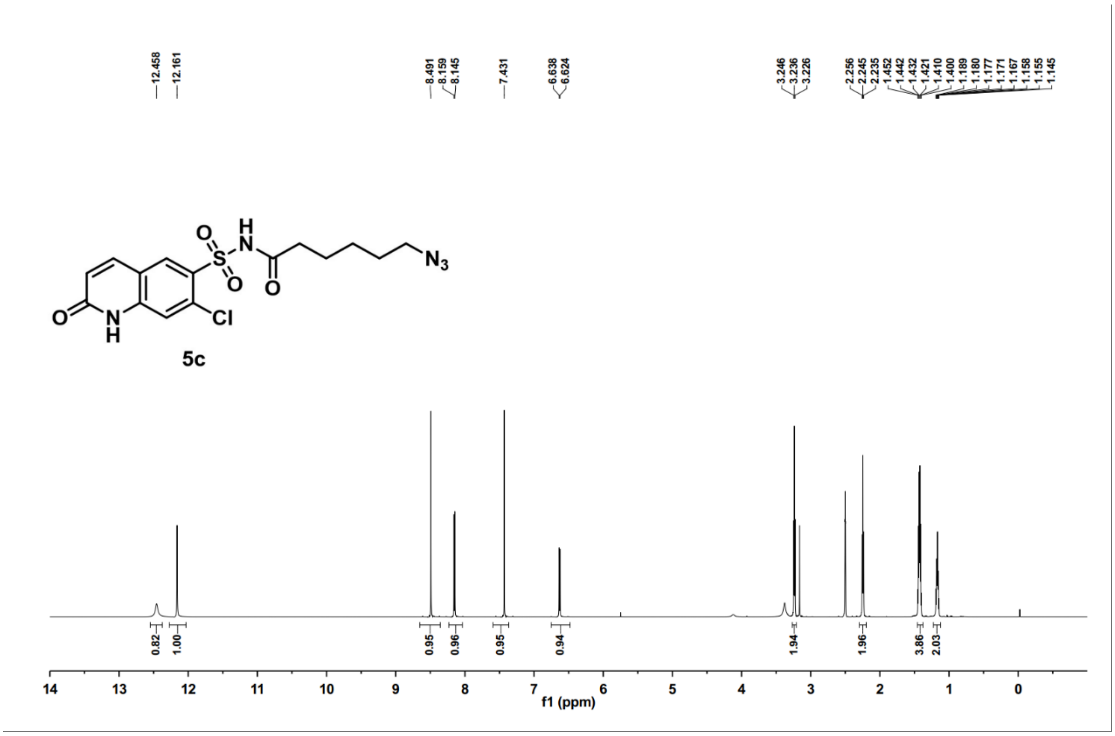

12.458
12.161
8.491
8.159
8.145
7.431
6.638
6.624
3.246
3.236
3.226
2.256
2.245
2.235
1.452
1.442
1.432
1.421
1.410
1.400
1.189
1.180
1.177
1.171
1.167
1.158
1.155
1.145
5c
0.82
1.00
0.95
0.96
0.95
0.94
1.94
1.96
3.86
2.03
f1 (ppm)


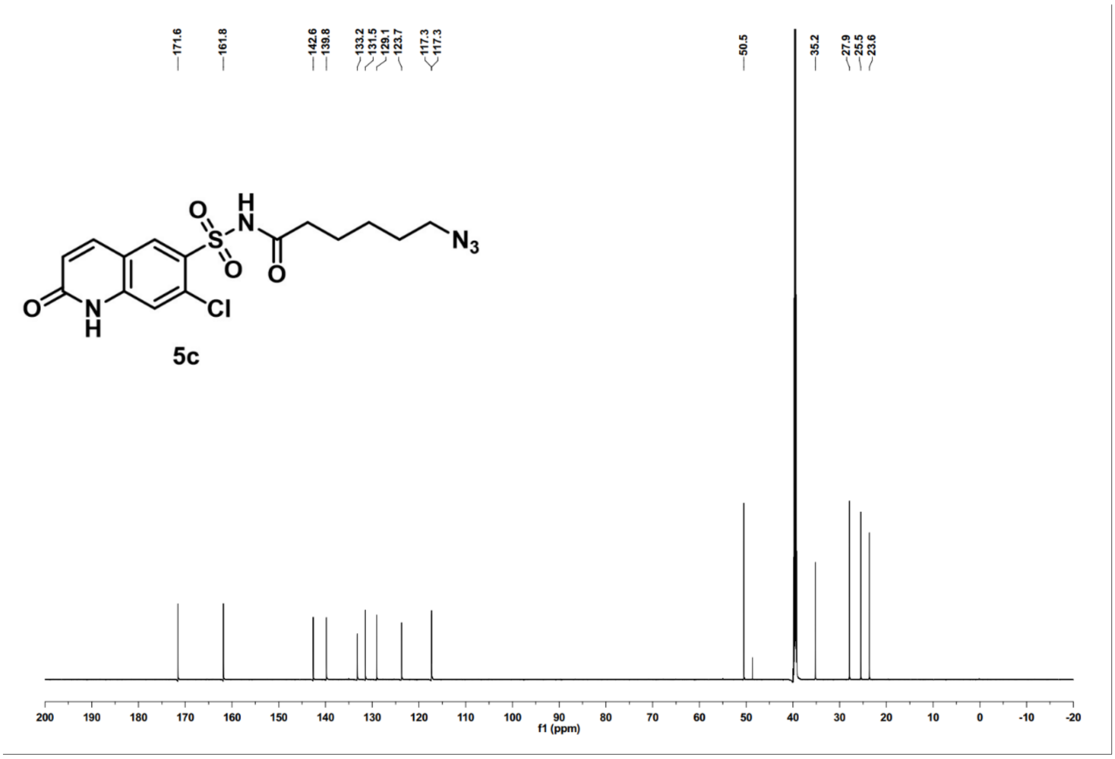

171.6
161.8
142.6
139.8
133.2
131.5
129.1
123.7
117.3
117.3
50.5
35.2
27.9
25.5
23.6
5c
f1 (ppm)

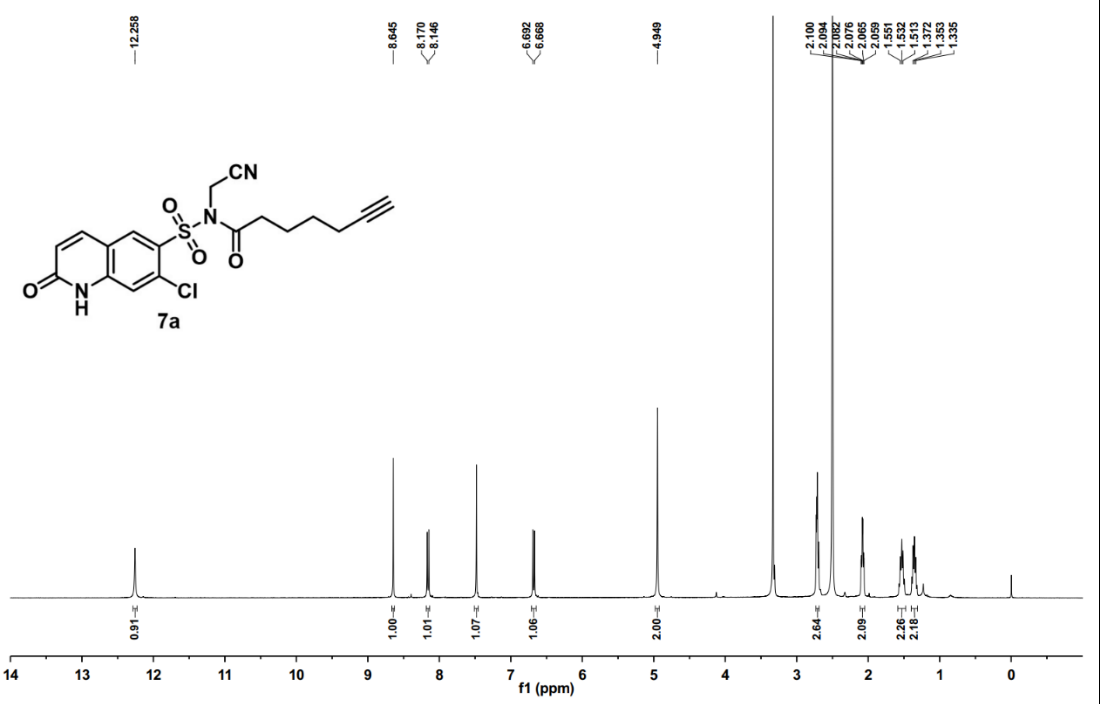
12.258
8.645
8.170
8.146
6.692
6.668
4.949
2.100
2.094
2.082
2.076
2.065
2.059
1.551
1.532
1.513
1.372
1.353
1.335
CN
O
S
N
O
O
O
N
H
Cl
7a
0.91
1.00
1.01
1.07
1.06
2.00
2.64
2.09
2.26
2.18
f1 (ppm)

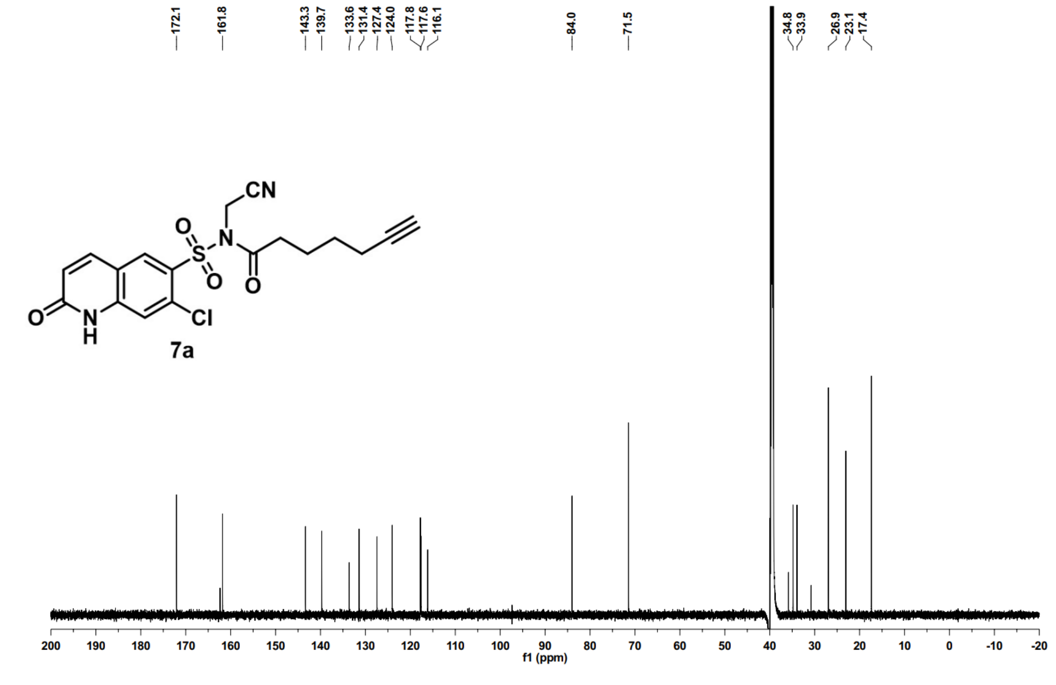
172.1
161.8
143.3
139.7
133.6
131.4
127.4
124.0
117.8
117.6
116.1
84.0
71.5
34.8
33.9
26.9
23.1
17.4
CN
O
S
N
O
O
O
N
H
Cl
7a
f1 (ppm)

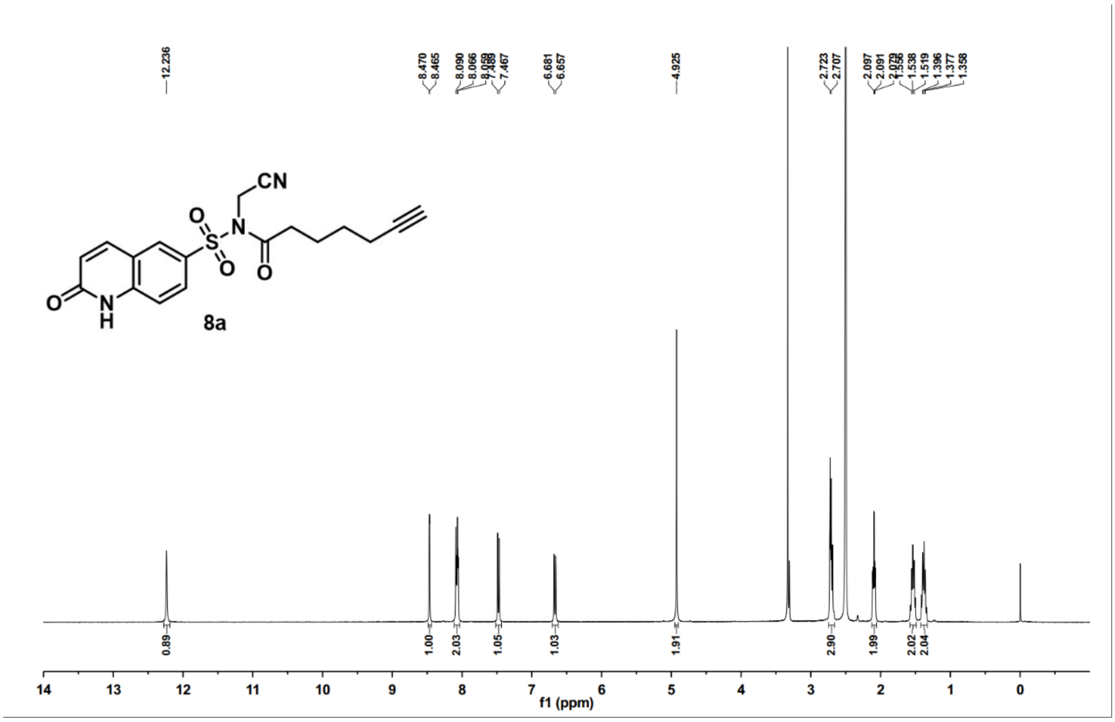
12.236
8.470
8.465
8.090
8.066
7.467
6.681
6.657
4.925
2.723
2.707
2.097
2.091
1.538
1.519
1.396
1.377
1.358
CN
O
S
N
O
O
O
N
H
8a
0.89
1.00
2.03
1.05
1.03
1.91
2.90
1.99
2.02
2.04
14 13 12 11 10 9 8 7 6 5 4 3 2 1 0
f1 (ppm)

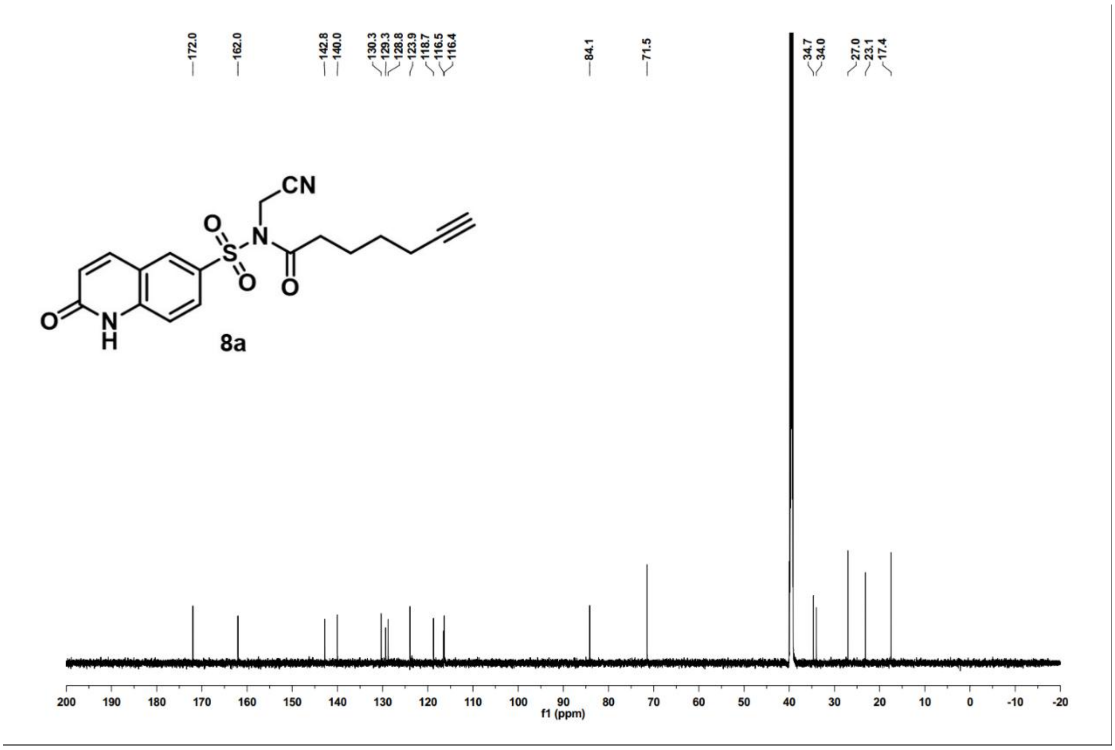
172.0
162.0
142.8
140.0
130.3
129.3
128.8
123.9
118.7
116.5
116.4
84.1
71.5
34.7
34.0
27.0
23.1
17.4
CN
O
S
N
O
O
O
N
H
8a
200 190 180 170 160 150 140 130 120 110 100 90 80 70 60 50 40 30 20 10 0 -10 -20
f1 (ppm)

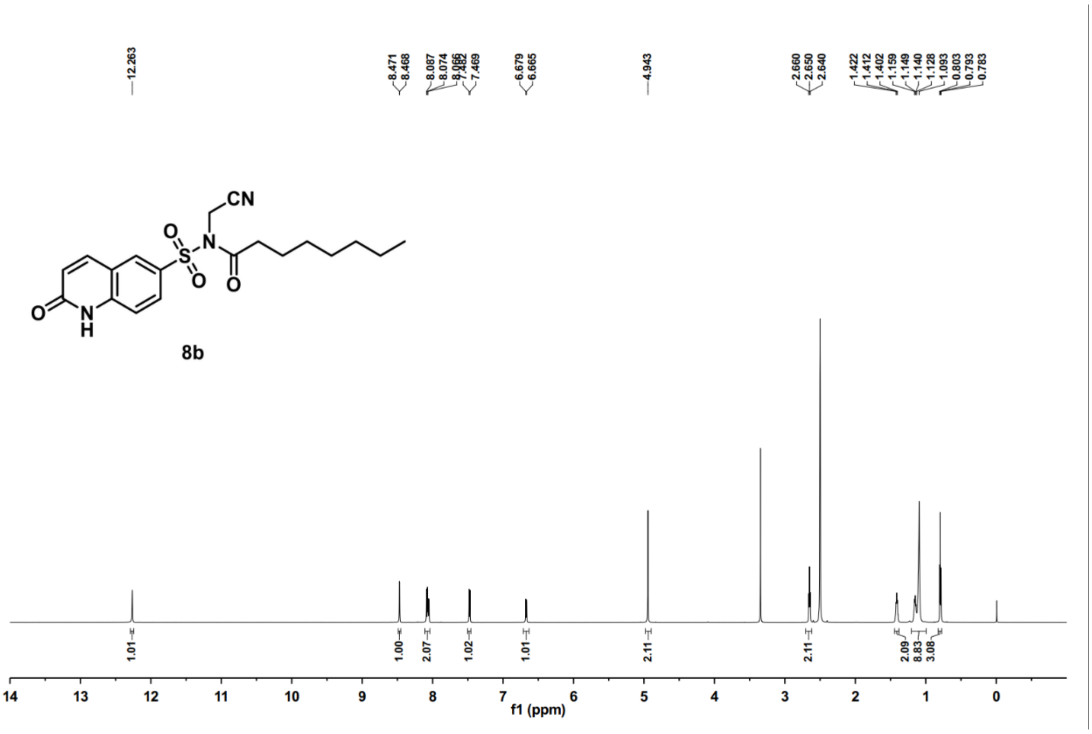
12.263
8.471
8.468
8.087
8.074
7.469
6.679
6.665
4.943
2.660
2.650
2.640
1.422
1.412
1.402
1.159
1.149
1.140
1.128
1.093
0.803
0.793
0.783
CN
N
S
O
O
O
O
N
H
8b
1.01
1.00
2.07
1.02
1.01
2.11
2.11
2.09
8.83
3.08
f1 (ppm)

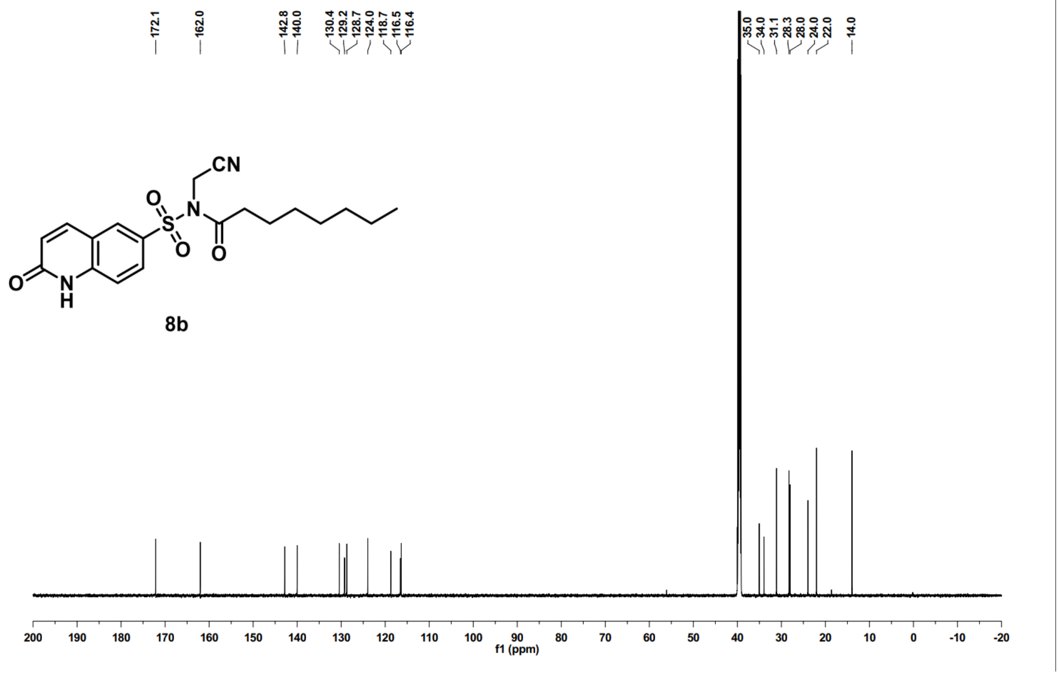
172.1
162.0
142.8
140.0
130.4
129.2
128.7
124.0
118.7
116.5
116.4
35.0
34.0
31.1
28.3
28.0
24.0
22.0
14.0
CN
N
S
O
O
O
O
N
H
8b
f1 (ppm)

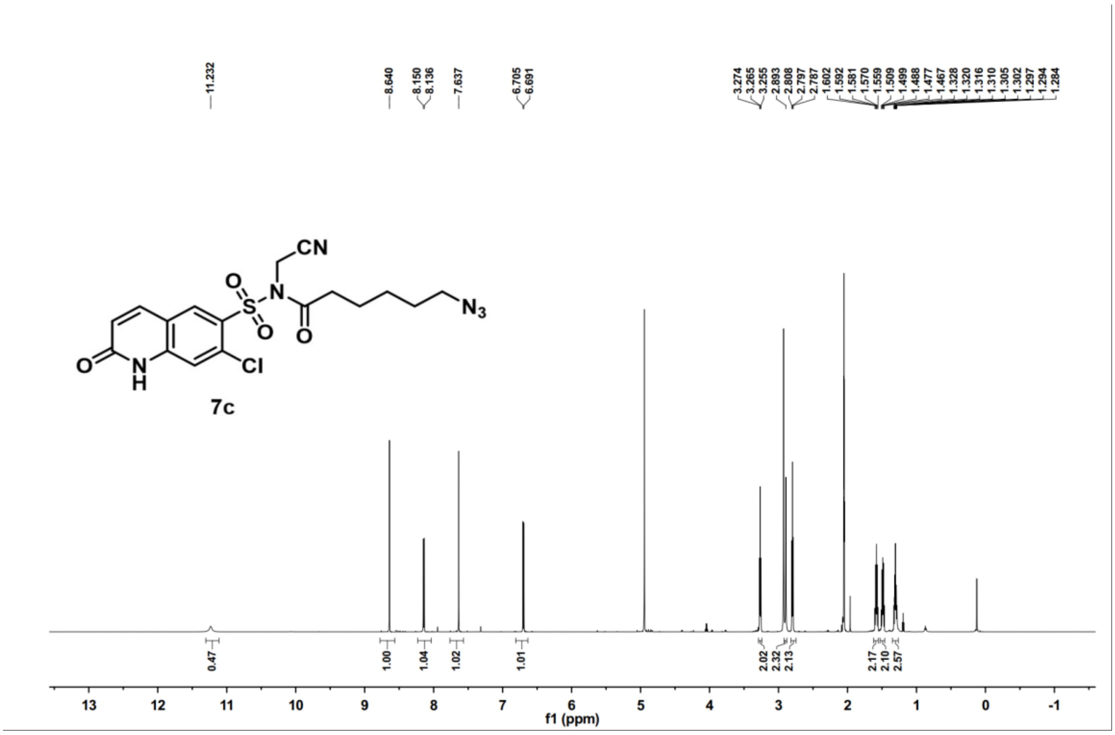

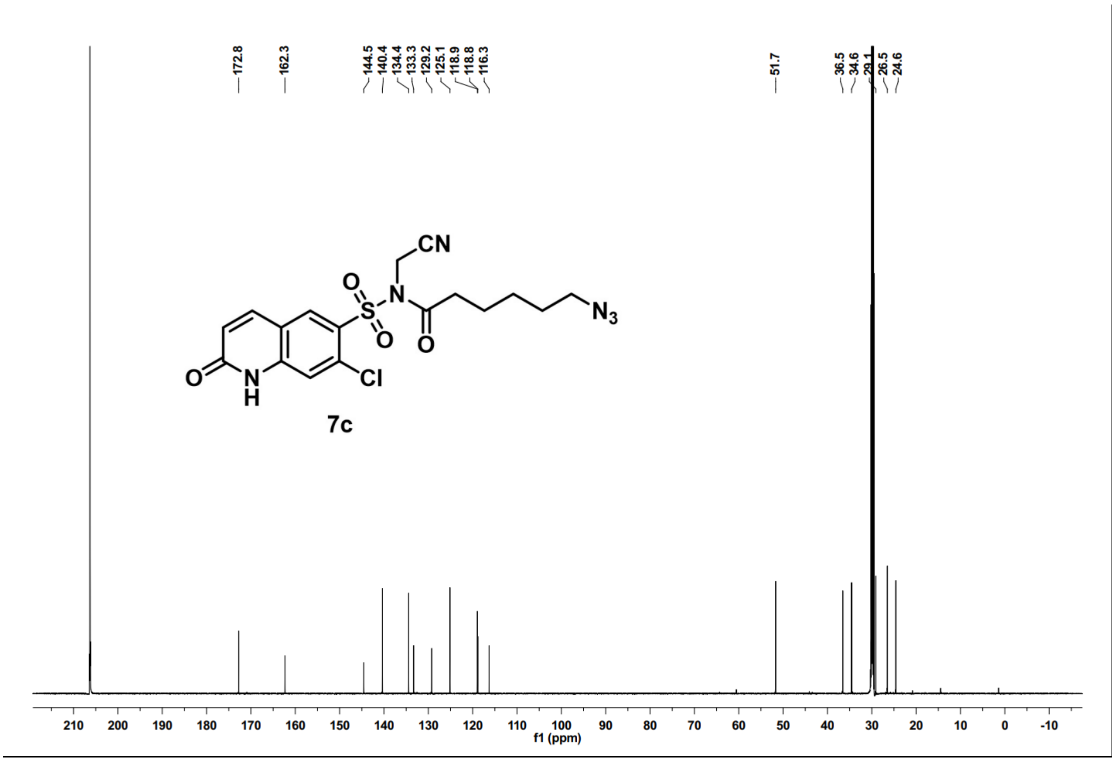

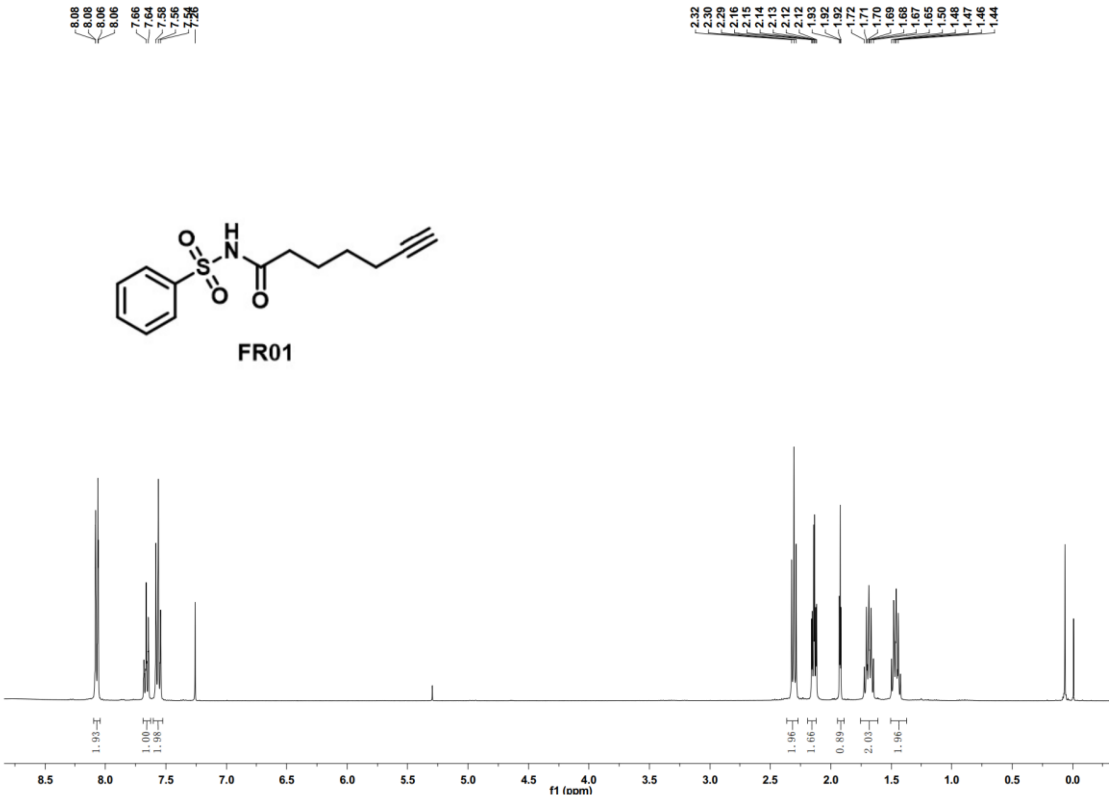
8.08
8.08
8.06
8.06
7.66
7.64
7.58
7.56
7.54
2.32
2.30
2.29
2.16
2.15
2.14
2.13
2.12
2.12
1.93
1.92
1.92
1.72
1.71
1.70
1.69
1.68
1.67
1.65
1.50
1.48
1.47
1.46
1.44
FR01
1.93
1.00
1.98
1.96
1.66
0.89
2.03
1.96
f1 (ppm)

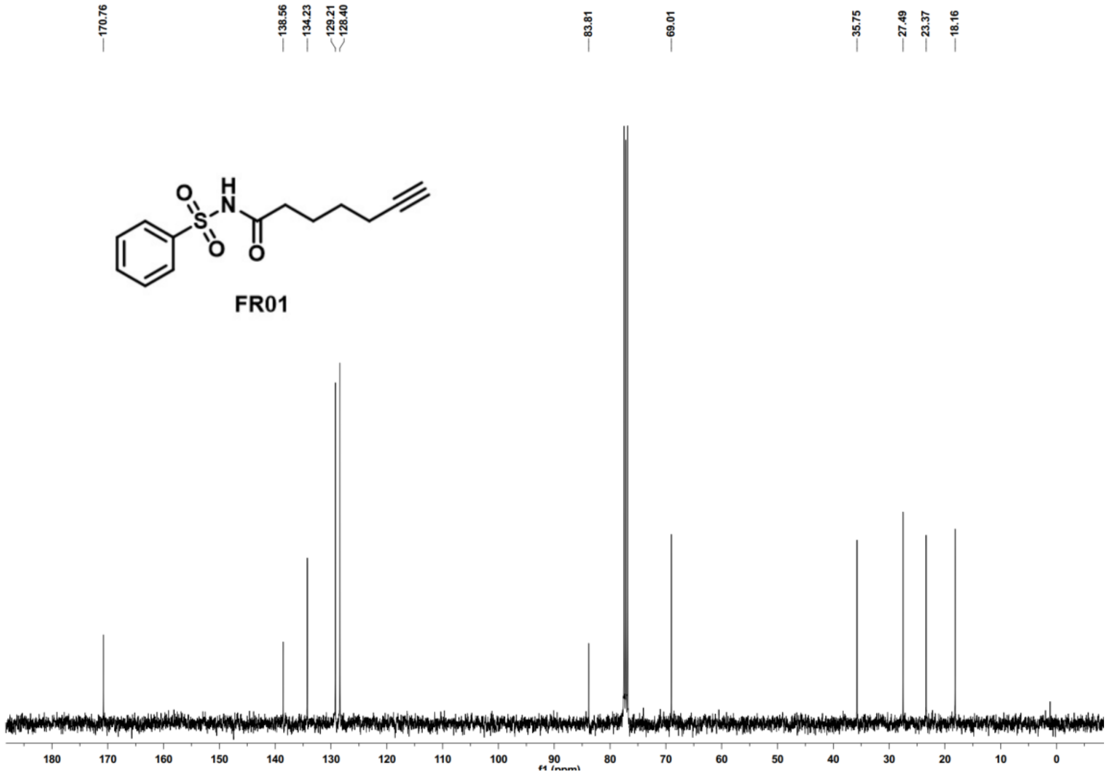
170.76
138.56
134.23
129.21
128.40
83.81
69.01
35.75
27.49
23.37
18.16
FR01
f1 (ppm)

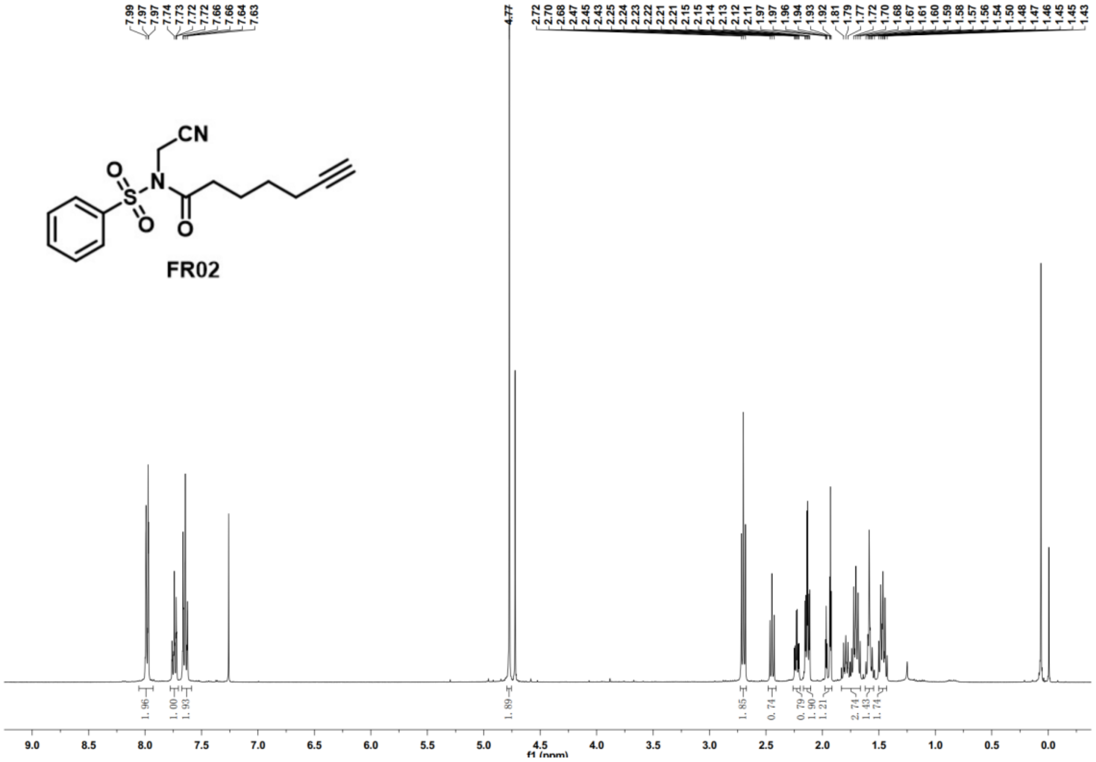

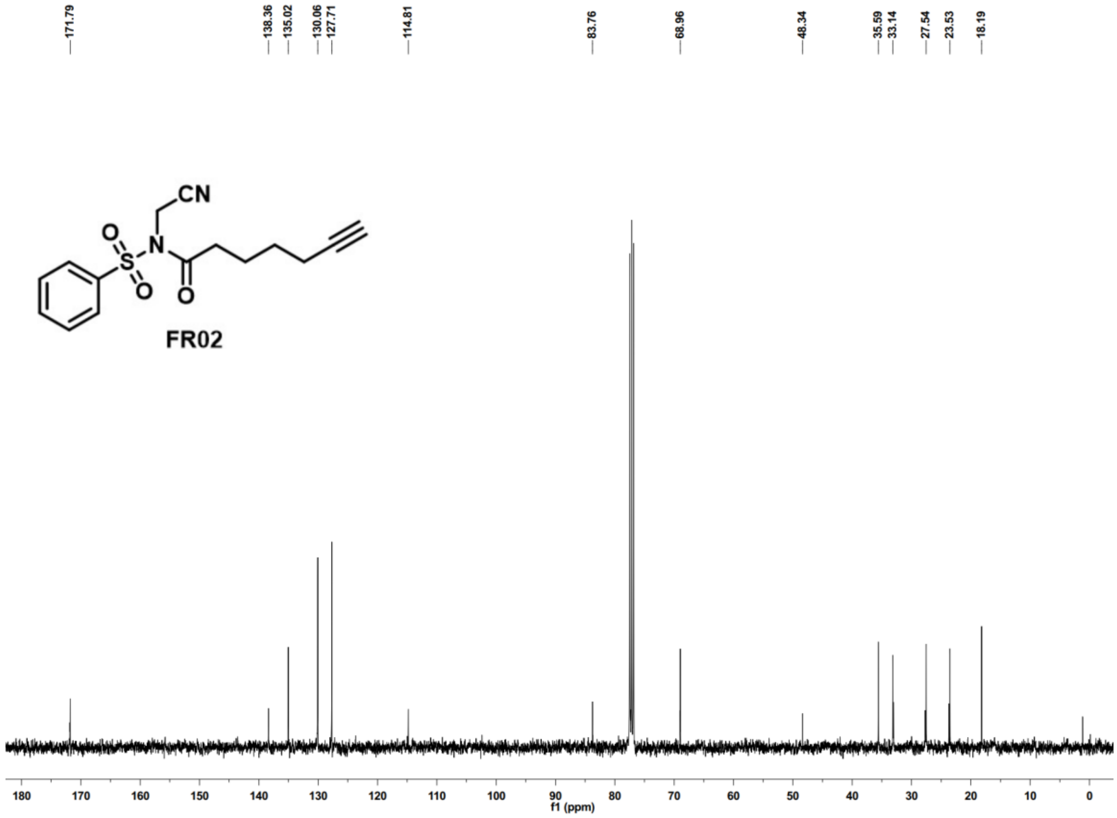

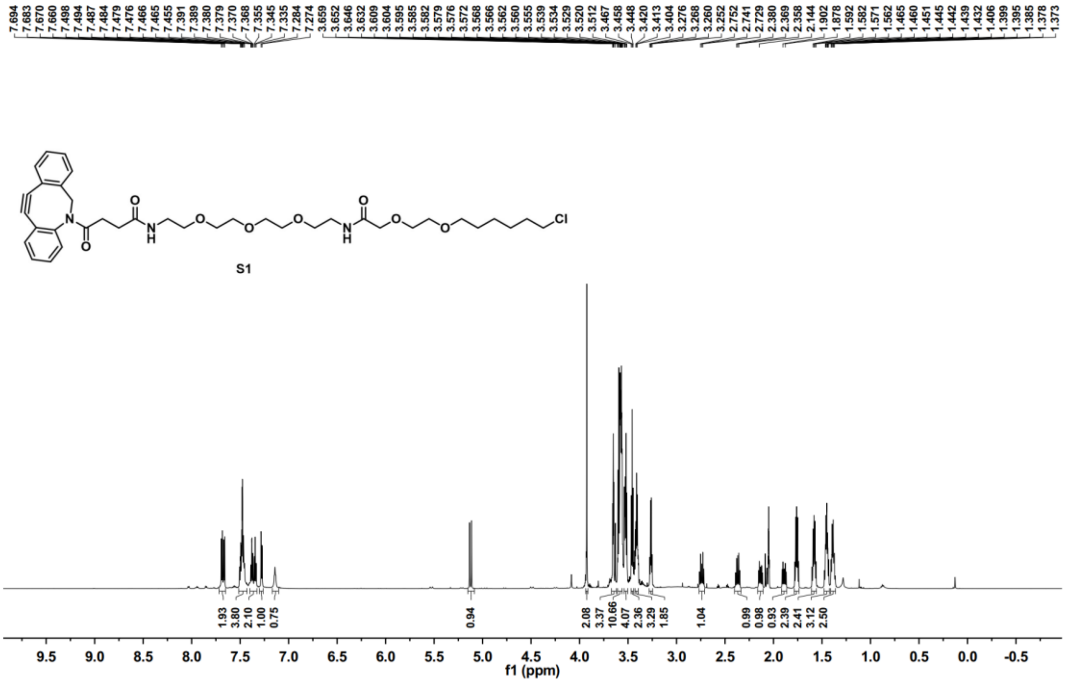
S1
f1 (ppm)

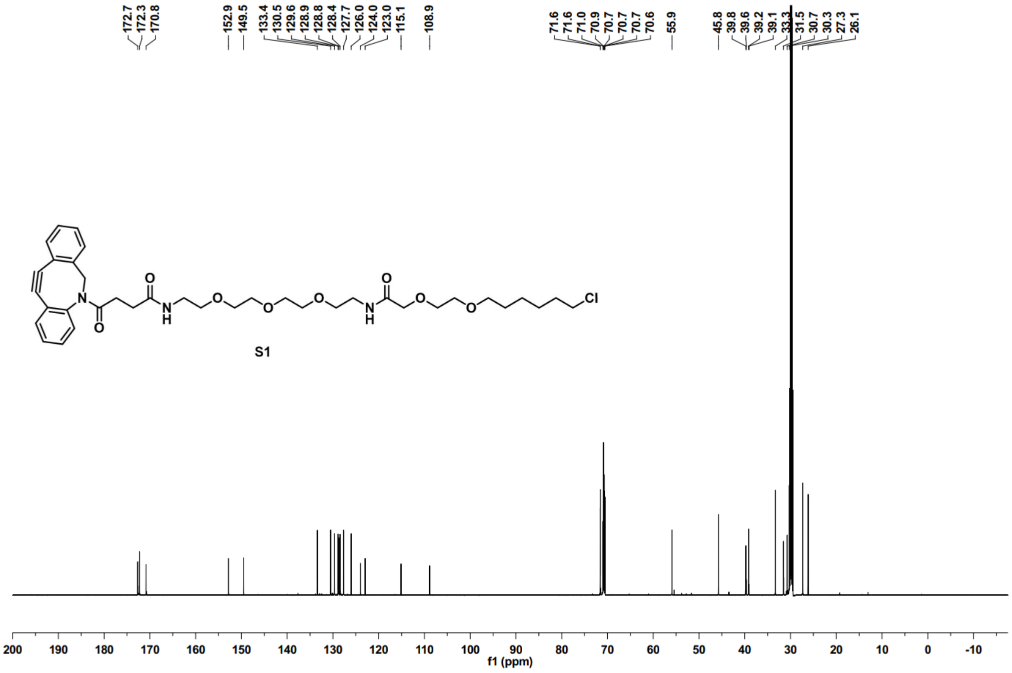
S1
f1 (ppm)
